# Supplementary material for: D-MannosE to prevent Recurrent urinary tract InfecTions (MERIT): protocol for a randomised controlled trial
Source: BMJ Open. 2021 Jan 13;11(1):e037128. doi: 10.1136/bmjopen-2020-037128 (PMC7812098; doi:10.1136/bmjopen-2020-037128)
Supplement: Supplementary data [file bmjopen-2020-037128supp001.pdf]

NUFFIELD DEPARTMENT OF PRIMARY CARE HEALTH SCIENCES

MERIT Informed Consent Form

Participant ID: __ __ __ - __ __

| | | PLEASE INITIAL |
|---|---|---|
| 1 | I confirm I have read and understood the information sheet V____ dated __ __ / __ __ __ / __ __ __ __ for the above study and have had the opportunity to ask questions and have these answered satisfactorily. | ___ ___ |
| 2 | I understand my participation is voluntary and that I am free to withdraw at any time, without giving any reason, and without my medical care or legal rights being affected. | ___ ___ |
| 3 | I understand that relevant sections of my medical notes and data collected during the study may be looked at by individuals from the University of Oxford, the relevant GP practice and from regulatory authorities, where it is relevant to my taking part in research. I give permission for these individuals to have access to my records. | ___ ___ |
| 4 | I agree for my identifiable information to be stored securely for up to three months after the end of the study, and research documents with my personal information (i.e. this signed Consent Form) stored securely for five years after the end of the study. | ___ ___ |
| 5 | I agree to my General Practitioner being informed of my participation in the study, and being sent a copy of this signed Consent Form. | ___ ___ |
| 6 | I agree to donate urine samples, which will be frozen and stored for further analyses relating to this study. I consider these samples a gift to the University of Oxford and I understand I will not gain any direct personal or financial benefit from them. | ___ ___ |
| 7 | I understand that I will be asked to complete questionnaires online using a website from the University of Oxford | ___ ___ |
| 8 | I agree to take part in this study. | ___ ___ |



Franssen M, *et al. BMJ Open* 2021; 11:e037128. doi: 10.1136/bmjopen-2020-037128

NUFFIELD DEPARTMENT OF PRIMARY CARE HEALTH SCIENCES — UNIVERSITY OF OXFORD

merit

MERIT Informed Consent Form

Participant ID: __ __ __ - __ __

Funded by NHS National Institute for Health Research

| ***ADDITIONAL (optional, not required for study participation)*** | **YES** | **NO** |
|---|---|---|
| *I agree to donate one perineal swab sample at the beginning of the study, which will be stored for further analyses relating to this study. I consider this sample a gift to the University of Oxford and I understand I will not gain any direct personal or financial benefit from this.* | __ __ | __ __ |
| *I agree to being contacted about the qualitative interviews in this trial* | __ __ | __ __ |
| *I agree to be contacted about ethically approved research studies for which I may be suitable. I agree that my identifiable information will be stored securely for five years after the end of the study. I understand that agreeing to be contacted does not oblige me to participate in any further studies.* | __ __ | __ __ |
| *I agree for my de-identified urine samples to be used in future research, here or abroad, which has ethical approval. I understand this research may involve commercial organisations.* | __ __ | __ __ |

**Participant:**

Signed: ______________________ Name: ______________________________ Date: __ __ / __ __ / __ __ __ __

**Researcher:**

Signed: ______________________ Name: _____________________________ Date: __ __ / __ __ / __ __ __ __



Franssen M, *et al. BMJ Open* 2021; 11:e037128. doi: 10.1136/bmjopen-2020-037128
