# Supplementary material for: D-MannosE to prevent Recurrent urinary tract InfecTions (MERIT): protocol for a randomised controlled trial
Source: BMJ Open. 2021 Jan 13;11(1):e037128. doi: 10.1136/bmjopen-2020-037128 (PMC7812098; doi:10.1136/bmjopen-2020-037128)
Supplement: Author's manuscript [file bmjopen-2020-037128.draft_revisions.pdf]

BMJ Open is committed to open peer review. As part of this commitment we make the peer review history of every article we publish publicly available.

When an article is published we post the peer reviewers' comments and the authors' responses online. We also post the versions of the paper that were used during peer review. These are the versions that the peer review comments apply to.

The versions of the paper that follow are the versions that were submitted during the peer review process. They are not the versions of record or the final published versions. They should not be cited or distributed as the published version of this manuscript.

BMJ Open is an open access journal and the full, final, typeset and author-corrected version of record of the manuscript is available on our site with no access controls, subscription charges or pay-per-view fees (http://bmjopen.bmj.com).

If you have any questions on BMJ Open's open peer review process please email info.bmjopen@bmj.com

BMJ Open

D-mannose to prevent recurrent urinary tract infections (MERIT): protocol for a randomised controlled trial.

Journal:	*BMJ Open*
Manuscript ID	bmjopen-2020-037128
Article Type:	Protocol
Date Submitted by the Author:	24-Jan-2020

Complete List of Authors:	Franssen, Marloes; University of Oxford, Nuffield Department of Orthopaedics, Rheumathology and Musculoskeletal Sciences<br>Cook, Johanna; University of Oxford, Nuffield Department of Primary Care Health Sciences<br>Robinson, Jared; University of Oxford, Nuffield Department of Primary Care Health Sciences<br>Williams, Nicola; Nuffield Department of Primary Care Health Sciences, University of Oxford<br>Glogowska, Margaret; University of Oxford, Nuffield Department of Primary Care Health Sciences<br>Yang, Yaling; University of Oxford, Nuffield Department of Primary Care Health Sciences<br>Allen, Julie; University of Oxford, Nuffield Department of Primary Care Health Sciences<br>Butler, Christopher C.; University of Oxford, Nuffield Department of Primary Care Health Sciences<br>Thomas, Nick; Windrush Medical Practice<br>Hay, Alastair; University of Bristol, Centre for Academic Primary Care<br>Moore, Michael; University of Southampton Medical School, Primary Care Medical Group<br>Hayward, Gail; University of Oxford, Nuffield Department of Primary Care Health Sciences

Keywords:	UROLOGY, Urinary tract infections < UROLOGY, QUALITATIVE RESEARCH

SCHOLARONE™
Manuscripts

I, the Submitting Author has the right to grant and does grant on behalf of all authors of the Work (as defined in the below author licence), an exclusive licence and/or a non-exclusive licence for contributions from authors who are: i) UK Crown employees; ii) where BMJ has agreed a CC-BY licence shall apply, and/or iii) in accordance with the terms applicable for US Federal Government officers or employees acting as part of their official duties; on a worldwide, perpetual, irrevocable, royalty-free basis to BMJ Publishing Group Ltd ("BMJ") its licensees and where the relevant Journal is co-owned by BMJ to the co-owners of the Journal, to publish the Work in this journal and any other BMJ products and to exploit all rights, as set out in our licence.

The Submitting Author accepts and understands that any supply made under these terms is made by BMJ to the Submitting Author unless you are acting as an employee on behalf of your employer or a postgraduate student of an affiliated institution which is paying any applicable article publishing charge ("APC") for Open Access articles. Where the Submitting Author wishes to make the Work available on an Open Access basis (and intends to pay the relevant APC), the terms of reuse of such Open Access shall be governed by a Creative Commons licence – details of these licences and which Creative Commons licence will apply to this Work are set out in our licence referred to above.

Other than as permitted in any relevant BMJ Author's Self Archiving Policies, I confirm this Work has not been accepted for publication elsewhere, is not being considered for publication elsewhere and does not duplicate material already published. I confirm all authors consent to publication of this Work and authorise the granting of this licence.

For peer review only

D-mannose to prevent recurrent urinary tract infections (MERIT): protocol for a randomised controlled trial.

Running Heading: MERIT Protocol paper

Trial registration: ISRCTN 13283516

Protocol version 5.0 (07November2019)

Funder: National Institute for Health Research (NIHR) School for Primary Care Research Grant (Grant Reference No: 385)

Sponsor: Oxford University (ctrg@admin.ox.ac.uk)

Authors:

Marloes Franssen[1], Johanna Cook[2], Jared Robinson[2], Nicola Williams[2], Margaret Glogowska[2], Yaling Yang[2], Julie Allen[2], Chris C. Butler[2], Nick Thomas[3], Alastair D Hay[4], Michael Moore[5], Gail Hayward[2]

1: Nuffield Department of Orthopaedics, Rheumathology and Musculoskeletal Sciences, Oxford University, United Kingdom

2: Nuffield Department of Primary Care, Oxford University, Oxford, United Kingdom

3: GP Principal, Windrush Medical Practice, Witney; Clinical Research Specialty Lead - Primary Care (NIHR Clinical Research Network: Thames Valley and South Midlands), United Kingdom

4: Centre for Academic Primary Care, NIHR School Primary Care Research, Bristol Medical School: Population Health Sciences, Bristol, United Kingdom

5: Primary Care, Population Sciences and Medical Education, Faculty of Medicine, University of Southampton, United Kingdom

Correspondence to: Dr Gail Hayward – gail.hayward@phc.ox.ac.uk

Word count: 2516 Intro to Discussion (excluding title page, abstract, references, tables and figures)

Number of tables: 1 Number of figures: 1

For peer review only

Abstract

Introduction: Recurrent urinary tract infections have a significant negative impact on quality of life and healthcare costs. To date, daily prophylactic antibiotics are the only treatment which have been shown to help prevent recurrent UTIs. D-mannose is a type of sugar which is believed to inhibit bacterial adherence to uroepithelial cells, and is already being used by some women in an attempt to prevent RUTIs. There is currently insufficient rigorous evidence on which to base decisions about its use. The MERIT study will evaluate whether D-mannose is clinically and cost effective in reducing frequency of infection and symptom burden for women presenting to UK primary care with recurrent UTI.

Methods and analysis: MERIT will be a two arm, individually randomised, double blind placebo controlled, pragmatic trial. Participants will be randomised to take D-mannose powder or placebo powder daily for six months. The primary outcome will be the number of medical attendances attributable to symptoms of UTI. With 508 participants we will have 90% power to detect a 50% reduction in the chance of a further clinically suspected UTI, assuming 20% loss to follow up. Secondary outcomes will include: number of days of moderately bad symptoms of UTI; time to next consultation; number of clinically suspected UTIs; number of microbiologically proven UTIs; number of antibiotic courses for UTI; quality of life and healthcare utilisation related to UTI. A within trial economic evaluation will be conducted to examine cost-effectiveness of D-mannose in comparison with placebo. A nested qualitative study will explore participants' experiences and perceptions of recruitment to, and participation in a study requiring a daily treatment.

Ethics and dissemination: Ethical approval has been obtained from South West – Central Bristol Research Ethics Committee. Publication of the MERIT study is anticipated to occur in 2021.

Trial Registration: ISRCTN 13283516

Keywords: UTI recurrence, RCT, Primary care, D-Mannose, cost effectiveness, qualitative interview

For peer review only

Strengths and limitations:

Strengths:

- Based on current literature this will be the first large publicly funded randomised controlled trial of D-mannose for prophylaxis of recurrent urinary tract infections.
- This study will fill a major gap in the evidence base about whether women with recurrent UTIs should initiate or continue to use this food supplement to prevent RUTI.

Weaknesses

- The trial may not be powered to detect a secondary outcome of symptom burden which is also of value to patient decision making
- Although participants report weekly on their study product usage there are no objective measures available to confirm accuracy of reporting

For peer review only

1. BACKGROUND

Urinary tract infection (UTI) is the most common bacterial infection that women consult for in UK primary care (1, 2). Approximately 40-50% of women experience one UTI episode during their lives (3). Recurrent UTIs (RUTIs) have a considerable negative impact on quality of life, which extends beyond the unpleasant symptoms to distressing and disrupted sexual relationships, persistent unmanageable pain and systemic illness (4). UTI accounts for an important proportion of health care costs as a result of outpatient visits, diagnostic tests and prescriptions (5). In 2007, UTI recurrence accounted for 10.5 million outpatient consultations and 2–3 million emergency department visits in the USA alone. In addition, UTIs are the most common cause of infection in hospitalized patients, accounting for 17.2% of all nosocomial infections in England. Furthermore, UTIs result in considerable patient morbidity and time off work; hence, the management of this condition incurs large financial costs, estimated at $3.5 billion in the USA per year (6).

A systematic review of randomised controlled trials identified antibiotic prophylaxis as the only treatment which has been demonstrated to help prevent RUTIs. Antibiotics taken daily for six to twelve months were more effective than placebo at preventing recurrent infection (7), and national guidelines advocate their use for this indication (8). However, antibiotics also resulted in more severe and unpleasant side effects (e.g. vomiting, urticaria, candidiasis). Furthermore, once antibiotic prophylaxis is discontinued, even after extended periods, approximately 50-60% of women will experience a further UTI within three months (9, 10). Thus, antibiotic prophylaxis does not exert benefit once stopped, and is directly linked to antibiotic resistance in uropathogens (11). Antibiotic resistance has been associated with an increased duration of severe symptoms of UTIs, irrespective of the use of an appropriate antibiotic (2, 11).

D-mannose is a type of sugar (a monosaccharide isomer of glucose), which is thought to inhibit bacterial adherence to uroepithelial cells by binding to a site on the tip of the fimbria (12) and has shown benefit in animal models in preventing UTI (13).

Currently D-mannose is available commercially to the public as a food supplement, and is favoured by many women who have RUTIs, but until recently, there has been little empirical evidence to support its use. An open label randomised three arm trial including 308 women with RUTI seen in outpatient settings (14) found that daily use of D-mannose for six months resulted in an absolute reduction in incidence of further UTI of 45% from a proportion of 60% in the usual care arm, with no adverse events. The proportion of women experiencing a UTI over six months was reduced by 11% compared to daily antibiotic use. This finding is supported by recent smaller studies (15-17).

Although there are indicators of efficacy from small underpowered trials, the only adequately powered study to date (15) was not placebo controlled and found an unexpectedly high UTI incidence in the control arm. Furthermore, a microbiologically confirmed UTI was a requirement for entry to the study, and participants were withdrawn once they developed a UTI on treatment, meaning true incidence of UTI could not be established, a measure for women who experience frequent UTIs, who are also the most likely candidates for prophylaxis. Finally, all women on hormonal contraception were excluded, which may reduce applicability to the women at high risk of RUTI.

For peer review only

D-mannose is found naturally in small quantities in numerous food sources, such as coffee, baker's yeast, egg white, fruits such as apples, cranberries and mangos, and also in legumes such as soybeans, kidney beans and peanuts (18). It is absorbed in the upper gastrointestinal tract and excreted in the urine (14).

D-mannose may offer an alternative to antibiotic prophylaxis in women who experience RUTI and in turn to contribute to better antimicrobial stewardship in primary care. However, the current evidence base is inadequate to help women with RUTI to make informed decisions about the use of D-mannose prophylaxis. The high costs (at least £25 a month) associated with its purchase add weight to the need to establish whether GPs should advise their patients to use D-mannose for this indication.

The MERIT double blind placebo-controlled randomised controlled trial aims to evaluate the effectiveness of D-mannose in women suffering with RUTI presenting to UK primary care and its cost effectiveness.

2. METHODS AND DESIGN

2.1 Study aims, research questions and outcomes

The primary aim of MERIT is to assess the effectiveness of daily use of D-mannose compared with placebo in preventing symptomatic UTI in women.

The primary outcome of the trial will be the proportion of women experiencing at least one further episode of clinically suspected UTI for which they contact ambulatory care (out of hours primary care, in hours primary care, ambulance or A&E) within six months of study entry.

Secondary outcomes will include (within six months of study entry):

-Number of days of moderately bad (or worse) symptoms of UTI

-Time to next consultation with a clinically suspected UTI

-Number of clinically suspected UTIs

-Number of microbiologically proven UTIs

-Number of antibiotic courses for UTI

-Report of consumption of antibiotics using diary during periods of infection

-Proportion of women with a resistant uropathogen culture during an episode of acute infection

-Hospital admissions related to UTI

-Quality of life and healthcare utilisation related to UTI

2.2 Study design and setting

A two arm, individually randomised, double blind placebo controlled, pragmatic trial.

For peer review only

2.3 Eligibility

This trial will recruit female participants over 18 years with a primary care clinical record of having presented to ambulatory care with RUTIs three or more times in the last year or two or more times in the last six months. Exclusion criteria are: participants who are pregnant, lactating or planning pregnancy during the course of the study; formal diagnosis of interstitial cystitis or overactive bladder syndrome; prophylactic antibiotics started in the last three months and unwilling to discontinue, or intention to start in the next six months; currently using D-mannose and unwilling to discontinue for the duration of the study; nursing home resident; catheterised, including intermittent self-catheterisation; use of Uromune; participation in a research study involving an investigational product in the past twelve weeks.

2.4 Baseline assessment

Participants will have a baseline assessment (either in person with their GP or research nurse online, or by telephone with a member of the research team). During the baseline assessment the study will be explained, informed consent will be obtained and data will be collected (see table 1). Participants will also be asked to send in a urine sample (when they are asymptomatic of a UTI) at baseline and have the option to also send in a perineal swab sample.

2.5 Randomisation

After the baseline assessment, participants will be randomised by a member of the research team using a validated internet based randomisation system with an emergency randomisation list available. Randomisation will be stratified by GP practice ensuring a balance of the two arms within each practice.

2.6 Intervention and placebo groups

Placebo will consist of two grams of a sugar powder which is similar in texture and taste to D-mannose but fully absorbed by the liver s to be taken daily for six months.

Intervention will consist of two grams of D-mannose powder to be taken daily for six months.

An adequate supply of the study product will be sent directly from the research team to the participant after randomisation, after two months and after four months.

2.7 Follow up

All participants will be asked to complete short weekly questionnaires, sent to them via text or email; they also will have the option of completing them telephonically directly with the research team. The weekly questionnaire will collect the information of participant’s adherence to study medication, and whether the participant has had any symptomatic UTI episodes. Participants will also be contacted monthly by phone by the research team to complete a monthly questionnaire which is similar to the weekly one if the weekly questionnaires are not being completed. The participants will be asked to complete a daily UTI symptom diary if they experience a UTI. The information will be collected via weekly and monthly contact if they fail to complete the symptom diary although they experience one. During a UTI they will be asked to send the lab a

For peer review only

urine sample, alongside any sample they might provide to their GP. They will also asked for a further urine sample two days after symptoms have resolved. See table 1 for details. Primary care electronic medical record reviews will be conducted to collect UTI related health care contacts, culture results, and prescriptions during the following up period. See figure 1 for the participant flow through the trial.

2.8 Sample size considerations

A recent study to evaluate prophylactic treatment for RUTI in a similar population (19) found that 26.6% of women in the control arm experienced a UTI within six months. Our patient and public involvement advisors suggested that in order to commit to daily use of a prophylactic regime, they would require evidence of at least a 50% reduction in the chance of a further UTI during the period of prophylaxis. To detect this reduction with 90% power and an alpha of 0.05 we would require 203 participants in each arm. This equates to 508 participants if a 20% loss to follow up is assumed. This sample size is also adequate to power the key secondary outcome (the number of UTI's experienced over six months), and detect a relative incidence rate of 0.5 between the treatment and placebo groups, assuming a base rate of 0.36 as estimated by Maki et al. (2016) (19). If the estimated percentage of participants who have either withdrawn or failed to respond to any study team communication for an extended period seems likely to rise above the 20% initially allowed for, we will recruit additional participants, up to a maximum of 598 participants.

2.9 Statistical analysis

The primary outcome, the proportion of women experiencing at least one further episode of UTI symptoms for which they visited their GP within six months of study entry, and other binary outcomes, will be analysed on an intention-to-treat basis by means of a generalised linear mixed effects model with binomial distribution and log link function, including a random effect for practice and fixed effect for randomisation group. Therefore, treatment groups will be compared on the basis of an adjusted risk ratio. The number of days of moderately bad symptoms of UTI, the number of UTI's experienced in six months, and number of antibiotic courses for UTI in six months, will be analysed by means of a generalised linear mixed effects model using the Poisson distribution and log link function, including a random effect for practice and a fixed effect for randomised group. Defined Daily Doses (DDDs) will be analysed by means of a linear mixed effects model including a random effect for practice and fixed effects for randomised group and baseline DDD, treating this outcome as continuous. We will analyse the overall DDD as well as the individual antibiotic DDDs.

2.10 Data management

Data Management will be performed in accordance with Primary Care Clinical Trials Unit Data Management standard operating procedures. Study specific procedures will be outlined in a Data Management Plan to ensure that high quality data are produced for statistical analysis.

2.11 Potential Risks

It is anticipated that the potential risks of this study are low and similar to those attributable to usual care.

2.12 Health economic evaluation

For peer review only

A cost effectiveness analysis (CEA) from a health system perspective with a time horizon of six months will be conducted alongside this study. The primary outcome measure for CUA will be the quality adjusted life years (QALYs). Data collection to facilitate analysis includes resource use and health outcomes. Data from the participant diary and electronic medical record review will be the main source of resource use. Unit costs associated with resource use items will be obtained from national standards. Health outcomes will be measured using the 5-level version of the EQ-5D questionnaire (EQ-5D-5L).

Data analysis will be conducted on an intention-to-treat basis using an incremental approach. Resource use and unit cost will be combined to calculate health care costs for each participant and mean cost for each study arm. EQ-5D-5L utility values will be calculated using the UK-based algorithm. Using the under the curve methods to combine utility values and associated time durations will produce QALYs for each participant and mean QALYs for each study arm during the six month study period. Mean differences in costs and QALYs between the study arms will be estimated as incremental cost per QALY gained. Given the fact that antibiotics are currently the mainstay treatment for both acute and recurrent UTIs, the issue of how the cost of antibiotic resistance should be incorporated into economic evaluation will be explored in the analysis.

2.13 Nested Qualitative study

We will recruit a maximum variation sample of 35 participants across both study arms for the nested qualitative study, continuing recruitment until data saturation is reached. A balanced list of participants will be drawn up for the qualitative researchers. The topic guide will include participants' experiences and perceptions of recruitment to, and participation in a study that requires taking a daily study product (whether D-mannose or placebo), exploring the level of perceived benefit patients anticipate would be required for them to continue this type of regime, and facilitators and barriers to adhering to prophylactic treatment. For participants' convenience, interviews will be conducted by telephone. Thematic analysis of the interviews will take into account issues identified from the literature and clinical research context, as well as inductively allowing new themes and ideas to emerge from the data. Analysis will be guided by the constant comparative method (20), which will include reading and familiarisation with the transcripts, noting and recording initial themes and then conducting systematic and detailed open coding using NVivo12 (21), a qualitative data analysis software. Analysis will proceed in an iterative manner – thus, the coding of a first set of interviews will generate an initial coding framework, which will be further developed and refined as further interviews are conducted and analysis proceeds. The researcher will draw on the clinical expertise of the rest of the research team in developing the coding framework and critically discussing ideas for categories emerging from the data, to ensure trustworthiness. A reflexive journal will assist in interpreting data and forming conclusions.

2.14 Patient and Public Involvement

Members of the public were involved in the design of the trial, reviewed patient facing documents and they will be active members of the trial steering committee.

3. DISCUSSION

The MERIT Study will be the first large, publicly funded, double blind randomised trial of the clinical and cost effectiveness of daily D-Mannose for preventing RUTI in primary care. This overview of the protocol describes

For peer review only

the plans for a pragmatic study recruiting women who suffer from recurrent UTI recruited in UK primary care. This study will fill a major gap in the evidence base about whether women with recurrent UTIs should initiate or continue to use this food supplement to prevent RUTI. If D-mannose is proven to be effective for the treatment of RUTIs this could benefit affected women and also contribute to antimicrobial stewardship. On the other hand, if found to be ineffective, costs spent on an ineffective intervention will be saved and attention can be refocussed on other, perhaps more effective prophylactic approaches, as well as redirected research efforts.

For peer review only

LIST OF ABBREVIATIONS

A&E: Accidents and Emergency; CUA: cost-utility analysis; DDD: defined daily dosage; EQ-5D-5L: 5 level version of the EQ-5D questionnaire; GP: General Practitioner; RUTIs: recurrent urinary tract infections; QALY: quality adjusted life years; UTI: Urinary Tract Infection;

DECLARATIONS

Ethics approval and consent to participate

Ethical approval has been obtained from South West – Central Bristol Research Ethics Committee (reference: 18/SW/0245). Any subsequent protocol amendments will be agreed with both sponsor and ethics committee prior to implementation. The study sponsor reviewed and ensured all indemnity and insurance requirements for the trial were in place prior to the start of recruitment. Participants will provide written informed consent prior to enrolment. Site specific approval has been obtained from the Thames Valley and South Midlands as a whole, and locally from all relevant Primary Care Organisations. An independent trial steering group will monitor study progress assisted by an independent data monitoring committee will periodically review the study.

Consent for publication

Not applicable

Availability of data and material

Not applicable as this is as study protocol and does not contain data or results.

Competing interests

None to declare

Funding

The trial is funded by a School for Primary Care research grant. Service support costs are administered through the NIHR Clinical Research Network: Thames Valley and South Midlands

This article presents independent research commissioned by the National Institute for Health Research (NIHR) under a School for Primary Care Research Grant (reference number:385). The views expressed in this publication are those of the authors and not necessarily those of the NHS, the NIHR or the Department of Health.

Authors contributions

GH had the original idea with CCB and they designed the study and obtained the funding. MF and JC wrote the first draft, NW provided the statistical section. YY provided the health economic section, MG provided the qualitative section. All authors subsequently critically edited the manuscript. GH will be guarantor for the manuscript.

For peer review only

Dissemination

The results of the MERIT will be published in peer-reviewed journals, in addition to being presented at conferences. It is anticipated that the results will be published in 2021, after the six month follow-up of all recruited participants.

Acknowledgements

The authors acknowledge the support of the Primary Care Clinical Trials Unit. Patient representatives are Sylvia Bailey and Valerie Tate. Additional members of the TSC are Rebecca Cannings-John (chair), Laura Shallcross and Akke Vellinga. The sponsor and funder had no role in the study design; collection, management, analysis, and interpretation of data, writing of the report; or the decision to submit the report for publication, which was made jointly by the authors who have all approved the final manuscript.

For peer review only

REFERENCES

1. Butler CC, Hillier S, Roberts Z, Dunstan F, Howard A, Palmer S. Antibiotic-resistant infections in primary care are symptomatic for longer and increase workload: outcomes for patients with E. coli UTIs. Br J Gen Pract. 2006;56(530):686-92.
2. Little P, Moore MV, Turner S, Rumsby K, Warner G, Lowes JA, et al. Effectiveness of five different approaches in management of urinary tract infection: randomised controlled trial. BMJ. 2010;340:c199.
3. Stapleton A. Host factors in susceptibility to urinary tract infections. Adv Exp Med Biol. 1999;462:351-8.
4. Flower A, Bishop FL, Lewith G. How women manage recurrent urinary tract infections: an analysis of postings on a popular web forum. BMC Fam Pract. 2014;15:162.
5. Butler CC, Hawking MK, Quigley A, McNulty CA. Incidence, severity, help seeking, and management of uncomplicated urinary tract infection: a population-based survey. Br J Gen Pract. 2015;65(639):e702-7.
6. Sihra N, Goodman A, Zakri R, Sahai A, Malde S. Nonantibiotic prevention and management of recurrent urinary tract infection. Nat Rev Urol. 2018;15(12):750-76.
7. Albert X, Huertas I, Pereiro, II, Sanfelix J, Gosalbes V, Perrota C. Antibiotics for preventing recurrent urinary tract infection in non-pregnant women. Cochrane Database Syst Rev. 2004(3):CD001209.
8. SIGN. Management of suspected bacterial urinary tract infection in adults 2012 [Available from: http://www.sign.ac.uk.
9. Car J, Sheikh A. Recurrent urinary tract infection in women. BMJ. 2003;327(7425):1204.
10. Harding GK, Ronald AR, Nicolle LE, Thomson MJ, Gray GJ. Long-term antimicrobial prophylaxis for recurrent urinary tract infection in women. Rev Infect Dis. 1982;4(2):438-43.
11. Costelloe C, Metcalfe C, Lovering A, Mant D, Hay AD. Effect of antibiotic prescribing in primary care on antimicrobial resistance in individual patients: systematic review and meta-analysis. BMJ. 2010;340:c2096.
12. Bouckaert J, Berglund J, Schembri M, De Genst E, Cools L, Wuhrer M, et al. Receptor binding studies disclose a novel class of high-affinity inhibitors of the Escherichia coli FimH adhesin. Mol Microbiol. 2005;55(2):441-55.
13. Michaels EK, Chmiel JS, Plotkin BJ, Schaeffer AJ. Effect of D-mannose and D-glucose on Escherichia coli bacteriuria in rats. Urol Res. 1983;11(2):97-102.
14. Sharma V, Ichikawa M, Freeze HH. Mannose metabolism: more than meets the eye. Biochem Biophys Res Commun. 2014;453(2):220-8.
15. Kranjcec B, Papes D, Altarac S. D-mannose powder for prophylaxis of recurrent urinary tract infections in women: a randomized clinical trial. World J Urol. 2014;32(1):79-84.
16. Xing Hu YS, Peng Zhang, Ming Miao, Toa Zhang, Bo Jiang. D-Mannose: Properties, Production, and Applications: An Overview. Comprehensive Reviews in Food Science and Food Safety. 2016;15:773-85.
17. Domenici L, Monti M, Bracchi C, Giorgini M, Colagiovanni V, Muzii L, et al. D-mannose: a promising support for acute urinary tract infections in women. A pilot study. Eur Rev Med Pharmacol Sci. 2016;20(13):2920-5.
18. Xing Hu ea. d-Mannose: Properties, production and Applications: An Overview.
19. Maki KC, Kaspar KL, Khoo C, Derrig LH, Schild AL, Gupta K. Consumption of a cranberry juice beverage lowered the number of clinical urinary tract infection episodes in women with a recent history of urinary tract infection. Am J Clin Nutr. 2016;103(6):1434-42.
20. Silverman DT. Doing Qualitative Research. Fifth Edition ed: Sage: London; 2017.
21. International Q. NVivo [Available from: https://www.qsrinternational.com/nvivo/what-is-nvivo/nvivo-12-promo.

For peer review only

Table 1: Data collection throughout the trial

Baseline:
1. Demographic questions: including age,
2. Past medical history [by patients]
3. Use of contraceptives and hormonal treatment
4. UTI episodes in the last 12 months
5. EQ-5D-5L
Weekly contact/Monthly contact:
1. UTI episodes in last week/month respectively
Daily UTI diary
1. UTI symptoms
2. UTI treatment
3. EQ-5D-5L
Six month questionnaire
1. UTI episodes in the last month
2. EQ-5D-5L
Notes review and urine culture result
1. Recorded UTIs during the study period
2. Healthcare contact for UTIs recorded
3. Antibiotics given for UTIs recorded
4. Culture results for UTIs recorded
5. Unscheduled hospital admissions

For peer review only

Figure 1: Flow through the trial

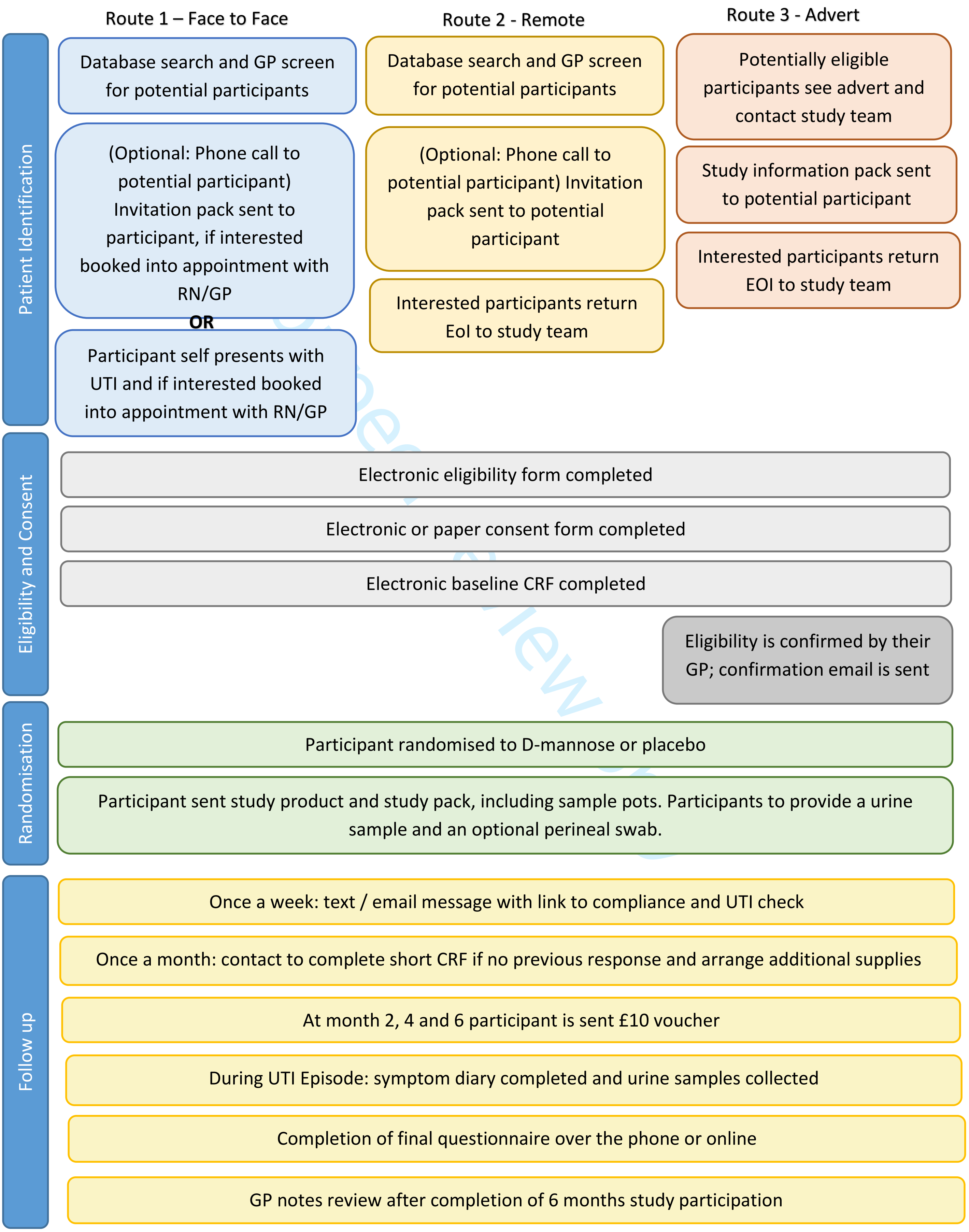

SPIRIT

STANDARD PROTOCOL ITEMS: RECOMMENDATIONS FOR INTERVENTIONAL TRIALS

SPIRIT 2013 Checklist: Recommended items to address in a clinical trial protocol and related documents*

Section/item	Item No	Description	Page Number on which item is reported
**Administrative information**			
Title	1	Descriptive title identifying the study design, population, interventions, and, if applicable, trial acronym	1
Trial registration	2a	Trial identifier and registry name. If not yet registered, name of intended registry	1
	2b	All items from the World Health Organization Trial Registration Data Set	1
Protocol version	3	Date and version identifier	1
Funding	4	Sources and types of financial, material, and other support	10
Roles and responsibilitie s	5a	Names, affiliations, and roles of protocol contributors	1
	5b	Name and contact information for the trial sponsor	1
	5c	Role of study sponsor and funders, if any, in study design; collection, management, analysis, and interpretation of data; writing of the report; and the decision to submit the report for publication, including whether they will have ultimate authority over any of these activities	14
	5d	Composition, roles, and responsibilities of the coordinating centre, steering committee, endpoint adjudication committee, data management team, and other individuals or groups overseeing the trial, if applicable (see Item 21a for data monitoring committee)	10
**Introduction**			

For peer review only

Background and rationale	6a	Description of research question and justification for undertaking the trial, including summary of relevant studies (published and unpublished) examining benefits and harms for each intervention	4
	6b	Explanation for choice of comparators	6
Objectives	7	Specific objectives or hypotheses	5
Trial design	8	Description of trial design including type of trial (eg, parallel group, crossover, factorial, single group), allocation ratio, and framework (eg, superiority, equivalence, noninferiority, exploratory)	5
**Methods: Participants, interventions, and outcomes**			
Study setting	9	Description of study settings (eg, community clinic, academic hospital) and list of countries where data will be collected. Reference to where list of study sites can be obtained	5
Eligibility criteria	10	Inclusion and exclusion criteria for participants. If applicable, eligibility criteria for study centres and individuals who will perform the interventions (eg, surgeons, psychotherapists)	6
Interventions	11a	Interventions for each group with sufficient detail to allow replication, including how and when they will be administered	6
	11b	Criteria for discontinuing or modifying allocated interventions for a given trial participant (eg, drug dose change in response to harms, participant request, or improving/worsening disease)	7
	11c	Strategies to improve adherence to intervention protocols, and any procedures for monitoring adherence (eg, drug tablet return, laboratory tests)	6
	11d	Relevant concomitant care and interventions that are permitted or prohibited during the trial	7
Outcomes	12	Primary, secondary, and other outcomes, including the specific measurement variable (eg, systolic blood pressure), analysis metric (eg, change from baseline, final value, time to event), method of aggregation (eg, median, proportion), and time point for each outcome. Explanation of the clinical relevance of chosen efficacy and harm outcomes is strongly recommended	5

For peer review only - http://bmjopen.bmj.com/site/about/guidelines.xhtml

Participant timeline	13	Time schedule of enrolment, interventions (including any run-ins and washouts), assessments, and visits for participants. A schematic diagram is highly recommended (see Figure)	6
Sample size	14	Estimated number of participants needed to achieve study objectives and how it was determined, including clinical and statistical assumptions supporting any sample size calculations	7
Recruitment	15	Strategies for achieving adequate participant enrolment to reach target sample size	7
**Methods: Assignment of interventions (for controlled trials)**			
Allocation:			
Sequence generation	16a	Method of generating the allocation sequence (eg, computer-generated random numbers), and list of any factors for stratification. To reduce predictability of a random sequence, details of any planned restriction (eg, blocking) should be provided in a separate document that is unavailable to those who enrol participants or assign interventions	6
Allocation concealment mechanism	16b	Mechanism of implementing the allocation sequence (eg, central telephone; sequentially numbered, opaque, sealed envelopes), describing any steps to conceal the sequence until interventions are assigned	6
Implementation	16c	Who will generate the allocation sequence, who will enrol participants, and who will assign participants to interventions	6
Blinding (masking)	17a	Who will be blinded after assignment to interventions (eg, trial participants, care providers, outcome assessors, data analysts), and how	5
	17b	If blinded, circumstances under which unblinding is permissible, and procedure for revealing a participant's allocated intervention during the trial	NA
**Methods: Data collection, management, and analysis**			

Data collection methods	18a	Plans for assessment and collection of outcome, baseline, and other trial data, including any related processes to promote data quality (eg, duplicate measurements, training of assessors) and a description of study instruments (eg, questionnaires, laboratory tests) along with their reliability and validity, if known. Reference to where data collection forms can be found, if not in the protocol	6
	18b	Plans to promote participant retention and complete follow-up, including list of any outcome data to be collected for participants who discontinue or deviate from intervention protocols	6
Data management	19	Plans for data entry, coding, security, and storage, including any related processes to promote data quality (eg, double data entry; range checks for data values). Reference to where details of data management procedures can be found, if not in the protocol	7
Statistical methods	20a	Statistical methods for analysing primary and secondary outcomes. Reference to where other details of the statistical analysis plan can be found, if not in the protocol	7
	20b	Methods for any additional analyses (eg, subgroup and adjusted analyses)	7
	20c	Definition of analysis population relating to protocol non-adherence (eg, as randomised analysis), and any statistical methods to handle missing data (eg, multiple imputation)	7
**Methods: Monitoring**			
Data monitoring	21a	Composition of data monitoring committee (DMC); summary of its role and reporting structure; statement of whether it is independent from the sponsor and competing interests; and reference to where further details about its charter can be found, if not in the protocol. Alternatively, an explanation of why a DMC is not needed	10
	21b	Description of any interim analyses and stopping guidelines, including who will have access to these interim results and make the final decision to terminate the trial	NA

Harms	22	Plans for collecting, assessing, reporting, and managing solicited and spontaneously reported adverse events and other unintended effects of trial interventions or trial conduct	NA
Auditing	23	Frequency and procedures for auditing trial conduct, if any, and whether the process will be independent from investigators and the sponsor	NA
**Ethics and dissemination**			
Research ethics approval	24	Plans for seeking research ethics committee/institutional review board (REC/IRB) approval	2
Protocol amendments	25	Plans for communicating important protocol modifications (eg, changes to eligibility criteria, outcomes, analyses) to relevant parties (eg, investigators, REC/IRBs, trial participants, trial registries, journals, regulators)	10
Consent or assent	26a	Who will obtain informed consent or assent from potential trial participants or authorised surrogates, and how (see Item 32)	6
	26b	Additional consent provisions for collection and use of participant data and biological specimens in ancillary studies, if applicable	NA
Confidentiality	27	How personal information about potential and enrolled participants will be collected, shared, and maintained in order to protect confidentiality before, during, and after the trial	NA
Declaration of interests	28	Financial and other competing interests for principal investigators for the overall trial and each study site	10
Access to data	29	Statement of who will have access to the final trial dataset, and disclosure of contractual agreements that limit such access for investigators	NA
Ancillary and post-trial care	30	Provisions, if any, for ancillary and post-trial care, and for compensation to those who suffer harm from trial participation	NA
Dissemination policy	31a	Plans for investigators and sponsor to communicate trial results to participants, healthcare professionals, the public, and other relevant groups (eg, via publication, reporting in results databases, or other data sharing arrangements), including any publication restrictions	2

For peer review only

	31b	Authorship eligibility guidelines and any intended use of professional writers	NA
	31c	Plans, if any, for granting public access to the full protocol, participant-level dataset, and statistical code	NA
**Appendices**			
Informed consent materials	32	Model consent form and other related documentation given to participants and authorised surrogates	NA
Biological specimens	33	Plans for collection, laboratory evaluation, and storage of biological specimens for genetic or molecular analysis in the current trial and for future use in ancillary studies, if applicable	NA

*It is strongly recommended that this checklist be read in conjunction with the SPIRIT 2013 Explanation & Elaboration for important clarification on the items. Amendments to the protocol should be tracked and dated. The SPIRIT checklist is copyrighted by the SPIRIT Group under the Creative Commons "Attribution-NonCommercial-NoDerivs 3.0 Unported" license.

For peer review only

BMJ Open

D-mannose to prevent recurrent urinary tract infections (MERIT): protocol for a randomised controlled trial.

Journal:	*BMJ Open*
Manuscript ID	bmjopen-2020-037128.R1
Article Type:	Protocol
Date Submitted by the Author:	16-Sep-2020

Complete List of Authors:	Franssen, Marloes; University of Oxford, Nuffield Department of Orthopaedics, Rheumathology and Musculoskeletal Sciences<br>Cook, Johanna; University of Oxford, Nuffield Department of Primary Care Health Sciences<br>Robinson, Jared; University of Oxford, Nuffield Department of Primary Care Health Sciences<br>Williams, Nicola; Nuffield Department of Primary Care Health Sciences, University of Oxford<br>Glogowska, Margaret; University of Oxford, Nuffield Department of Primary Care Health Sciences<br>Yang, Yaling; University of Oxford, Nuffield Department of Primary Care Health Sciences<br>Allen, Julie; University of Oxford, Nuffield Department of Primary Care Health Sciences<br>Butler, Christopher C.; University of Oxford, Nuffield Department of Primary Care Health Sciences<br>Thomas, Nick; Windrush Medical Practice<br>Hay, Alastair; University of Bristol, Centre for Academic Primary Care<br>Moore, Michael; University of Southampton Medical School, Primary Care Medical Group<br>Hayward, Gail; University of Oxford, Nuffield Department of Primary Care Health Sciences

**Primary Subject Heading**:	Urology
Secondary Subject Heading:	General practice / Family practice, Health economics, Infectious diseases
Keywords:	UROLOGY, Urinary tract infections < UROLOGY, QUALITATIVE RESEARCH

SCHOLARONE™ Manuscripts

I, the Submitting Author has the right to grant and does grant on behalf of all authors of the Work (as defined in the below author licence), an exclusive licence and/or a non-exclusive licence for contributions from authors who are: i) UK Crown employees; ii) where BMJ has agreed a CC-BY licence shall apply, and/or iii) in accordance with the terms applicable for US Federal Government officers or employees acting as part of their official duties; on a worldwide, perpetual, irrevocable, royalty-free basis to BMJ Publishing Group Ltd ("BMJ") its licensees and where the relevant Journal is co-owned by BMJ to the co-owners of the Journal, to publish the Work in this journal and any other BMJ products and to exploit all rights, as set out in our licence.

The Submitting Author accepts and understands that any supply made under these terms is made by BMJ to the Submitting Author unless you are acting as an employee on behalf of your employer or a postgraduate student of an affiliated institution which is paying any applicable article publishing charge ("APC") for Open Access articles. Where the Submitting Author wishes to make the Work available on an Open Access basis (and intends to pay the relevant APC), the terms of reuse of such Open Access shall be governed by a Creative Commons licence – details of these licences and which Creative Commons licence will apply to this Work are set out in our licence referred to above.

Other than as permitted in any relevant BMJ Author's Self Archiving Policies, I confirm this Work has not been accepted for publication elsewhere, is not being considered for publication elsewhere and does not duplicate material already published. I confirm all authors consent to publication of this Work and authorise the granting of this licence.

For peer review only

D-mannose to prevent recurrent urinary tract infections (MERIT): protocol for a randomised controlled trial.

Running Heading: MERIT Protocol paper

Trial registration: ISRCTN 13283516

Protocol version 6.0 (17July2020)

Funder: National Institute for Health Research (NIHR) School for Primary Care Research Grant (Grant Reference No: 385)

Sponsor: Oxford University (ctrg@admin.ox.ac.uk)

Authors:

Marloes Franssen[1], Johanna Cook[2], Jared Robinson[2], Nicola Williams[2], Margaret Glogowska[2], Yaling Yang[2], Julie Allen[2], Chris C. Butler[2], Nick Thomas[3], Alastair D Hay[4], Michael Moore[5], Gail Hayward[2]

1: Nuffield Department of Orthopaedics, Rheumatology and Musculoskeletal Sciences, Oxford University, United Kingdom

2: Nuffield Department of Primary Care, Oxford University, Oxford, United Kingdom

3: GP Principal, Windrush Medical Practice, Witney; Clinical Research Specialty Lead - Primary Care (NIHR Clinical Research Network: Thames Valley and South Midlands), United Kingdom

4: Centre for Academic Primary Care, NIHR School Primary Care Research, Bristol Medical School: Population Health Sciences, Bristol, United Kingdom

5: Primary Care, Population Sciences and Medical Education, Faculty of Medicine, University of Southampton, United Kingdom

Correspondence to: Professor Gail Hayward – gail.hayward@phc.ox.ac.uk

Word count: 2685 Introduction to Discussion (excluding title page, abstract, references, tables and figures)

Number of tables: 1 Number of figures: 1

For peer review only

Abstract

Introduction: Recurrent urinary tract infections (RUTIs) have a significant negative impact on quality of life and healthcare costs. To date, daily prophylactic antibiotics are the only treatment which have been shown to help prevent RUTIs. D-mannose is a type of sugar which is believed to inhibit bacterial adherence to uroepithelial cells, and is already being used by some women in an attempt to prevent RUTIs. There is currently insufficient rigorous evidence on which to base decisions about its use. The MERIT study will evaluate whether D-mannose is clinically and cost effective in reducing frequency of infection and symptom burden for women presenting to UK primary care with recurrent UTI.

Methods and analysis: MERIT will be a two arm, individually randomised, double blind placebo controlled, pragmatic trial. Participants will be randomised to take D-mannose powder or placebo powder daily for six months. The primary outcome will be the number of medical attendances attributable to symptoms of RUTI. With 508 participants we will have 90% power to detect a 50% reduction in the chance of a further clinically suspected UTI, assuming 20% loss to follow up. Secondary outcomes will include: number of days of moderately bad symptoms of UTI; time to next consultation; number of clinically suspected UTIs; number of microbiologically proven UTIs; number of antibiotic courses for UTI; quality of life and healthcare utilisation related to UTI. A within trial economic evaluation will be conducted to examine cost-effectiveness of D-mannose in comparison with placebo. A nested qualitative study will explore participants' experiences and perceptions of recruitment to, and participation in a study requiring a daily treatment.

Ethics and dissemination: Ethical approval has been obtained from South West – Central Bristol Research Ethics Committee. Publication of the MERIT study is anticipated to occur in 2021.

Trial Registration: ISRCTN 13283516

Keywords: UTI recurrence, RCT, Primary care, D-Mannose, cost effectiveness, qualitative interview

For peer review only

Strengths and limitations:

- Based on current literature this will be the first large publicly funded randomised controlled trial of D-mannose for prophylaxis of recurrent urinary tract infections.
- This study is the first to use a placebo control in evaluating the benefit of D-mannose
- Obtaining the primary outcome by medical notes review will ensure data completeness
- The trial may not be powered to detect a secondary outcome of symptom burden which is also of value to patient decision making
- Although participants report weekly on their study product usage there are no objective measures available to confirm accuracy of reporting

For peer review only

1. BACKGROUND

Urinary tract infection (UTI) is the most common bacterial infection that women consult for in UK primary care (1, 2). Approximately 40-50% of women experience one UTI episode during their lives (3). Recurrent UTIs (RUTIs) have a considerable negative impact on quality of life, which extends beyond the unpleasant symptoms to distressing and disrupted sexual relationships, persistent unmanageable pain and systemic illness (4). UTI accounts for an important proportion of health care costs as a result of outpatient visits, diagnostic tests and prescriptions (5). In 2007, UTI recurrence accounted for 10.5 million outpatient consultations and 2–3 million emergency department visits in the USA alone. In addition, UTIs are the most common cause of infection in hospitalized patients, accounting for 17.2% of all nosocomial infections in England. Furthermore, UTIs result in considerable patient morbidity and time off work; hence, the management of this condition incurs large financial costs, estimated at $3.5 billion in the USA per year (6).

A systematic review of randomised controlled trials identified antibiotic prophylaxis as the only treatment which has been demonstrated to help prevent RUTIs. Antibiotics taken daily for six to twelve months were more effective than placebo at preventing recurrent infection (7), and national guidelines advocate their use for this indication (8). However, antibiotics also resulted in more severe and unpleasant side effects (e.g. vomiting, urticaria, candidiasis). Furthermore, once antibiotic prophylaxis is discontinued, even after extended periods, approximately 50-60% of women will experience a further UTI within three months (9, 10). Thus, antibiotic prophylaxis does not exert benefit once stopped, and is directly linked to antibiotic resistance in uropathogens (11). Antibiotic resistance has been associated with an increased duration of severe symptoms of UTIs, irrespective of the use of an appropriate antibiotic (2, 11).

D-mannose is a type of sugar (a monosaccharide isomer of glucose), which is thought to inhibit bacterial adherence to uroepithelial cells by binding to a site on the tip of the fimbria (12) and has shown benefit in animal models in preventing UTIs (13).

Currently D-mannose is available commercially to the public as a food supplement, and is favoured by many women who have RUTIs, but until recently, there has been little empirical evidence to support its use. An open label randomised three arm trial including 308 women with RUTI seen in outpatient settings (14) found that daily use of D-mannose for six months resulted in an absolute reduction in incidence of further UTI of 45% from a proportion of 60% in the usual care arm, with no adverse events. The proportion of women experiencing a RUTI over six months was reduced by 11% compared to daily antibiotic use. This finding is supported by recent smaller studies (15-18).

Although there are indicators of efficacy from small underpowered trials, the only adequately powered study to date (15) was not placebo controlled and found an unexpectedly high RUTI incidence in the control arm. Furthermore, a microbiologically confirmed UTI was a requirement for entry to the study, and participants were withdrawn once they developed a UTI on treatment. Therefore, true incidence of UTI could not be established, a measure for women who experience frequent RUTIs, who are also the most likely candidates for prophylaxis. Finally, all women on hormonal contraception were excluded, which may reduce applicability to the women at high risk of RUTI.

For peer review only

D-mannose is found naturally in small quantities in numerous food sources, such as coffee, baker's yeast, egg white, fruits such as apples, cranberries and mangos, and also in legumes such as soybeans, kidney beans and peanuts (19). It is absorbed in the upper gastrointestinal tract and excreted in the urine (14).

D-mannose may offer an alternative to antibiotic prophylaxis in women who experience RUTI and in turn to contribute to better antimicrobial stewardship in primary care. However, the current evidence base is inadequate to help women with RUTI to make informed decisions about the use of D-mannose prophylaxis. The high costs (at least £25 a month) associated with its purchase add weight to the need to establish whether GPs should advise their patients to use D-mannose for this indication.

The MERIT double blind placebo-controlled randomised controlled trial aims to evaluate the effectiveness of D-mannose in women suffering with RUTI presenting to UK primary care and its cost effectiveness.

2. METHODS AND DESIGN

2.1 Study aims, research questions and outcomes

The primary aim of MERIT is to assess the effectiveness of daily use of D-mannose compared with placebo in preventing symptomatic UTI in women.

The primary outcome of the trial will be the proportion of women experiencing at least one further episode of clinically suspected UTI for which they contact ambulatory care (out of hours primary care, in hours primary care, ambulance or the emergency department) within six months of study entry.

Secondary outcomes will include (within six months of study entry):

-Number of days of moderately bad (or worse) symptoms of UTI

-Time to next consultation with a clinically suspected UTI

-Number of clinically suspected UTIs

-Number of microbiologically proven UTIs

-Number of antibiotic courses for UTI

-Report of consumption of antibiotics using diary during periods of infection

-Proportion of women with a resistant uropathogen culture during an episode of acute infection

-Hospital admissions related to UTI

-Quality of life and healthcare utilisation related to UTI

-Healthcare utilisation recorded in the participant diary and during a notes review

-Acceptability and process evaluation conducted via telephone interviews (after 6 months)

For peer review only

2.2 Study design and setting

A two arm, individually randomised, double blind placebo controlled, pragmatic trial. At least 50 GP practices in England and Wales will be invited to take part in the trial. Recruitment will run from March 2019 until January 2020.

2.3 Eligibility

This trial will recruit female participants over 18 years with a primary care clinical record of having presented to ambulatory care with RUTIs three or more times in the last year or two or more times in the last six months. Exclusion criteria are: participants who are pregnant, lactating or planning pregnancy during the course of the study; formal diagnosis of interstitial cystitis or overactive bladder syndrome; prophylactic antibiotics started in the last three months and unwilling to discontinue, or intention to start in the next six months; currently using D-mannose and unwilling to discontinue for the duration of the study; nursing home resident; catheterised, including intermittent self-catheterisation; use of Uromune; participation in a research study involving an investigational product in the past twelve weeks.

2.4 Baseline assessment

Participants will have a baseline assessment (either in person with their GP or research nurse online, or by telephone with a member of the research team). During the baseline assessment the study will be explained, informed consent will be obtained and data will be collected (see table 1). Participants will also be asked to send in a urine sample (when they are asymptomatic of a UTI) at baseline and have the option to also send in a perineal swab sample.

Table 1: Data collection throughout the trial

Baseline:
1. Demographic questions: including age,
2. Past medical history [by patients]
3. Use of contraceptives and hormonal treatment
4. UTI episodes in the last 12 months
5. EQ-5D-5L
Weekly contact/Monthly contact:
1. UTI episodes in last week/month respectively
Daily UTI diary
1. UTI symptoms
2. UTI treatment
3. EQ-5D-5L

For peer review only

Six month questionnaire
1. UTI episodes in the last month
2. EQ-5D-5L
Notes review and urine culture result
1. Recorded UTIs during the study period
2. Healthcare contact for UTIs recorded
3. Antibiotics given for UTIs recorded
4. Culture results for UTIs recorded
5. Unscheduled hospital admissions

2.5 Randomisation

After the baseline assessment, participants will be randomised by a member of the research team using a validated internet based randomisation system with an emergency randomisation list available. Randomisation will use variable block sizes and will be stratified by GP practice ensuring a balance of the two arms within each practice.

2.6 Intervention and placebo groups

Placebo will consist of two grams of a sugar powder which is similar in texture and taste to D-mannose but fully absorbed by the liver to be taken daily for six months.

Intervention will consist of two grams of D-mannose powder to be taken daily for six months.

An adequate supply of the study product will be sent directly from the research team to the participant after randomisation, after two months and after four months.

2.7 Follow up

All participants will be asked to complete short weekly questionnaires, sent to them via text or email; they also will have the option of completing them telephonically directly with the research team. The weekly questionnaire will collect the information of participant's adherence to study medication, and whether the participant has had any symptomatic UTI episodes. Participants will also be contacted monthly by phone by the research team to complete a monthly questionnaire which is similar to the weekly one if there are two or more weekly questionnaires not being completed. They will also be asked to complete a daily UTI symptom diary if they experience a UTI. The information will be collected via weekly and monthly contact if they fail to complete the symptom diary although they experience one. During a UTI they will be asked to send the lab a urine sample, alongside any sample they might provide to their GP. They will also be asked for a further urine sample two days after symptoms have resolved. See table 1 for details. Primary care electronic medical record reviews will be conducted to collect UTI related health care contacts, culture results, and prescriptions during

For peer review only

the following up period. Participants will receive a £10 voucher after every two months of participation (£30 pounds in total). See figure 1 for the participant flow through the trial.

2.8 Sample size considerations

A recent study to evaluate prophylactic treatment for RUTI in a similar population (20) found that 26.6% of women in the control arm experienced a RUTI within six months. Our patient and public involvement advisors suggested that in order to commit to daily use of a prophylactic regime, they would require evidence of at least a 50% reduction in the chance of a further UTI during the period of prophylaxis. To detect this reduction with a 2-sided Fisher's Exact test with 90% power and an alpha of 0.05 we would require 203 participants in each arm. This equates to 508 participants if a 20% loss to follow up is assumed. This sample size is also adequate to power the key secondary outcome (the number of RUTI's experienced over six months), and detect a relative incidence rate of 0.5 between the treatment and placebo groups, assuming a base rate of 0.36 as estimated by Maki et al. (2016) (20). If the estimated percentage of participants who have either withdrawn or failed to respond to any study team communication for an extended period seems likely to rise above the 20% initially allowed for, we will recruit additional participants, up to a maximum of 598 participants.

2.9 Statistical analysis

The primary outcome, the proportion of women experiencing at least one further episode of UTI symptoms for which they visited their GP within six months of study entry, and other binary outcomes, will be analysed on an intention-to-treat basis by means of a generalised linear mixed effects model with binomial distribution and log link function, including a random effect for practice and fixed effect for randomisation group. Therefore, treatment groups will be compared on the basis of an adjusted risk ratio. The number of days of moderately bad symptoms of UTI, the number of UTI's experienced in six months, and number of antibiotic courses for UTI in six months, will be analysed by means of a generalised linear mixed effects model using the Poisson distribution and log link function, including a random effect for practice and a fixed effect for randomised group. Defined Daily Doses (DDDs) will be analysed by means of a linear mixed effects model including a random effect for practice and fixed effects for randomised group and baseline DDD, treating this outcome as continuous. We will analyse the overall DDD as well as the individual antibiotic DDDs.

The amount of missing primary outcome data is expected to be very low as it is collected via notes review. The model chosen to analyse the primary outcome implicitly accounts for data missing at random, however the data missing mechanism will be explored

2.10 Data management

Data Management will be performed in accordance with Primary Care Clinical Trials Unit Data Management standard operating procedures. Study specific procedures will be outlined in a Data Management Plan to ensure that high quality data are produced for statistical analysis.

2.11 Potential Risks

It is anticipated that the potential risks of this study are low and similar to those attributable to usual care.

For peer review only

2.12 Health economic evaluation

A cost effectiveness analysis from a health system perspective with a time horizon of six months will be conducted alongside this study. The primary outcome measure for the cost utility analysis will be the quality adjusted life years (QALYs). Data collection to facilitate analysis includes resource use and health outcomes. Data from the participant diary and electronic medical record review will be the main source of resource use. Unit costs associated with resource use items will be obtained from national standards. Health outcomes will be measured using the 5-level version of the EQ-5D questionnaire (EQ-5D-5L).

Data analysis will be conducted on an intention-to-treat basis using an incremental approach. Resource use and unit cost will be combined to calculate health care costs for each participant and mean cost for each study arm. EQ-5D-5L utility values will be calculated using the UK-based algorithm. Using the under the curve methods to combine utility values and associated time durations will produce QALYs for each participant and mean QALYs for each study arm during the six month study period. Mean differences in costs and QALYs between the study arms will be estimated as incremental cost per QALY gained. Given the fact that antibiotics are currently the mainstay treatment for both acute and recurrent UTIs, the issue of how the cost of antibiotic resistance should be incorporated into economic evaluation will be explored in the analysis.

2.13 Nested Qualitative study

We will recruit a maximum variation sample of 35 participants across both study arms for the nested qualitative study, continuing recruitment until data saturation is reached. A balanced list of participants will be drawn up for the qualitative researchers. The topic guide will include participants' experiences and perceptions of recruitment to, and participation in a study that requires taking a daily study product (whether D-mannose or placebo), exploring the level of perceived benefit patients anticipate would be required for them to continue this type of regimem, and facilitators and barriers to adhering to prophylactic treatment. For participants' convenience, interviews will be conducted by telephone. Thematic analysis of the interviews will take into account issues identified from the literature and clinical research context, as well as inductively allowing new themes and ideas to emerge from the data. Analysis will be guided by the constant comparative method (21), which will include reading and familiarisation with the transcripts, noting and recording initial themes and then conducting systematic and detailed open coding using NVivo12 (22), a qualitative data analysis software. Analysis will proceed in an iterative manner – thus, the coding of a first set of interviews will generate an initial coding framework, which will be further developed and refined as further interviews are conducted and analysis proceeds. The researcher will draw on the clinical expertise of the rest of the research team in developing the coding framework and critically discussing ideas for categories emerging from the data, to ensure trustworthiness. A reflexive journal will assist in interpreting data and forming conclusions.

2.14 Patient and Public Involvement

Members of the public were involved in the design of the trial, reviewed patient facing documents and they will be active members of the trial steering committee.

3. DISCUSSION

For peer review only

The MERIT Study will be the first large, publicly funded, double blind randomised trial of the clinical and cost effectiveness of daily D-Mannose for preventing RUTI in primary care. This overview of the protocol describes the plans for a pragmatic study recruiting women who suffer from recurrent UTI recruited in UK primary care. This study will fill a major gap in the evidence base about whether women with recurrent UTIs should initiate or continue to use this food supplement to prevent RUTI. If D-mannose is proven to be effective for the treatment of RUTIs this could benefit affected women and also contribute to antimicrobial stewardship. On the other hand, if found to be ineffective, costs spent on an ineffective intervention will be saved and attention can be refocussed on other, perhaps more effective prophylactic approaches, as well as redirected research efforts.

Supplementary files:

- Figure 1: Participant flow through the trial
- Example of participant consent form

LIST OF ABBREVIATIONS

DDD: defined daily dosage; EQ-5D-5L: 5 level version of the EQ-5D questionnaire; GP: General Practitioner; RUTIs: recurrent urinary tract infections; QALY: quality adjusted life years; UTI: Urinary Tract Infection;

DECLARATIONS

Ethics approval and consent to participate

Ethical approval has been obtained from South West – Central Bristol Research Ethics Committee (reference: 18/SW/0245). Any subsequent protocol amendments will be agreed with both sponsor and ethics committee prior to implementation. The study sponsor reviewed and ensured all indemnity and insurance requirements for the trial were in place prior to the start of recruitment. Participants will provide written informed consent prior to enrolment. Site specific approval has been obtained from the Thames Valley and South Midlands as a whole, and locally from all relevant Primary Care Organisations. An independent trial steering group will monitor study progress assisted by an independent data monitoring committee will periodically review the study.

Consent for publication

Not applicable

Availability of data and material

Not applicable as this is as study protocol and does not contain data or results.

Competing interests

None to declare

Funding

The trial is funded by a School for Primary Care research grant. Service support costs are administered through

For peer review only

the NIHR Clinical Research Network: Thames Valley and South Midlands

This article presents independent research commissioned by the National Institute for Health Research (NIHR) under a School for Primary Care Research Grant (reference number:385). The views expressed in this publication are those of the authors and not necessarily those of the NHS, the NIHR or the Department of Health.

Authors contributions

GH had the original idea with CCB and they designed the study and obtained the funding. MF and JC wrote the first draft, NW provided the statistical section. YY provided the health economic section, MG provided the qualitative section. All authors (JR, JA, NT, AH, MM) subsequently critically edited the manuscript. GH will be guarantor for the manuscript.

Dissemination

The results of the MERIT will be published in peer-reviewed journals, in addition to being presented at conferences. It is anticipated that the results will be published in 2021, after the six month follow-up of all recruited participants.

Acknowledgements

The authors acknowledge the support of the Primary Care Clinical Trials Unit. Patient representatives are Sylvia Bailey and Valerie Tate. Additional members of the TSC are Rebecca Cannings-John (chair), Laura Shallcross and Akke Vellinga. The sponsor and funder had no role in the study design; collection, management, analysis, and interpretation of data, writing of the report; or the decision to submit the report for publication, which was made jointly by the authors who have all approved the final manuscript.

For peer review only

REFERENCES

1. Butler CC, Hillier S, Roberts Z, Dunstan F, Howard A, Palmer S. Antibiotic-resistant infections in primary care are symptomatic for longer and increase workload: outcomes for patients with E. coli UTIs. Br J Gen Pract. 2006;56(530):686-92.
2. Little P, Moore MV, Turner S, Rumsby K, Warner G, Lowes JA, et al. Effectiveness of five different approaches in management of urinary tract infection: randomised controlled trial. BMJ. 2010;340:c199.
3. Stapleton A. Host factors in susceptibility to urinary tract infections. Adv Exp Med Biol. 1999;462:351-8.
4. Flower A, Bishop FL, Lewith G. How women manage recurrent urinary tract infections: an analysis of postings on a popular web forum. BMC Fam Pract. 2014;15:162.
5. Butler CC, Hawking MK, Quigley A, McNulty CA. Incidence, severity, help seeking, and management of uncomplicated urinary tract infection: a population-based survey. Br J Gen Pract. 2015;65(639):e702-7.
6. Sihra N, Goodman A, Zakri R, Sahai A, Malde S. Nonantibiotic prevention and management of recurrent urinary tract infection. Nat Rev Urol. 2018;15(12):750-76.
7. Albert X, Huertas I, Pereiro, II, Sanfelix J, Gosalbes V, Perrota C. Antibiotics for preventing recurrent urinary tract infection in non-pregnant women. Cochrane Database Syst Rev. 2004(3):CD001209.
8. SIGN. Management of suspected bacterial urinary tract infection in adults 2012 [Available from: http://www.sign.ac.uk.
9. Car J, Sheikh A. Recurrent urinary tract infection in women. BMJ. 2003;327(7425):1204.
10. Harding GK, Ronald AR, Nicolle LE, Thomson MJ, Gray GJ. Long-term antimicrobial prophylaxis for recurrent urinary tract infection in women. Rev Infect Dis. 1982;4(2):438-43.
11. Costelloe C, Metcalfe C, Lovering A, Mant D, Hay AD. Effect of antibiotic prescribing in primary care on antimicrobial resistance in individual patients: systematic review and meta-analysis. BMJ. 2010;340:c2096.
12. Bouckaert J, Berglund J, Schembri M, De Genst E, Cools L, Wuhrer M, et al. Receptor binding studies disclose a novel class of high-affinity inhibitors of the Escherichia coli FimH adhesin. Mol Microbiol. 2005;55(2):441-55.
13. Michaels EK, Chmiel JS, Plotkin BJ, Schaeffer AJ. Effect of D-mannose and D-glucose on Escherichia coli bacteriuria in rats. Urol Res. 1983;11(2):97-102.
14. Sharma V, Ichikawa M, Freeze HH. Mannose metabolism: more than meets the eye. Biochem Biophys Res Commun. 2014;453(2):220-8.
15. Kranjcec B, Papes D, Altarac S. D-mannose powder for prophylaxis of recurrent urinary tract infections in women: a randomized clinical trial. World J Urol. 2014;32(1):79-84.
16. Xing Hu YS, Peng Zhang, Ming Miao, Toa Zhang, Bo Jiang. D-Mannose: Properties, Production, and Applications: An Overview. Comprehensive Reviews in Food Science and Food Safety. 2016;15:773-85.
17. Domenici L, Monti M, Bracchi C, Giorgini M, Colagiovanni V, Muzii L, et al. D-mannose: a promising support for acute urinary tract infections in women. A pilot study. Eur Rev Med Pharmacol Sci. 2016;20(13):2920-5.
18. Kuzmenko AV, Kuzmenko VV, Gyaurgiev TA. [Efficacy of combined antibacterial-prebiotic therapy in combination with D-mannose in women with uncomplicated lower urinary tract infection]. Urologiia. 2019(6):38-43.
19. Xing Hu ea. d-Mannose: Properties, production and Applications: An Overview.
20. Maki KC, Kaspar KL, Khoo C, Derrig LH, Schild AL, Gupta K. Consumption of a cranberry juice beverage lowered the number of clinical urinary tract infection episodes in women with a recent history of urinary tract infection. Am J Clin Nutr. 2016;103(6):1434-42.
21. Silverman DT. Doing Qualitative Research. Fifth Edition ed: Sage: London; 2017.

For peer review only

22. International Q. NVivo [Available from: https://www.qsrinternational.com/nvivo/what-is-nvivo/nvivo-12-promo.

For peer review only

Figure 1: Flow through the trial

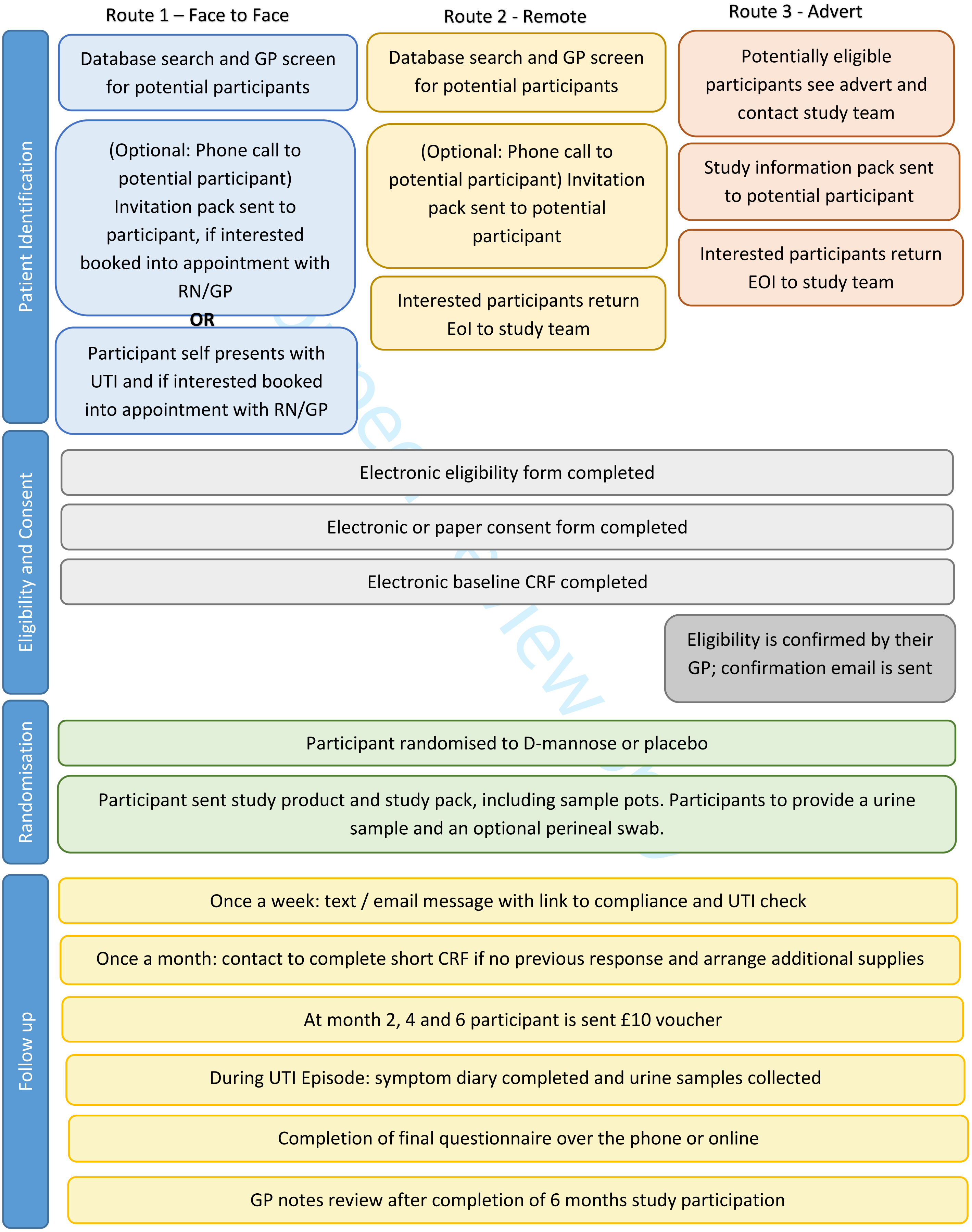

For peer review only - http://bmjopen.bmj.com/site/about/guidelines.xhtml

NUFFIELD DEPARTMENT OF PRIMARY CARE HEALTH SCIENCES
UNIVERSITY OF OXFORD

merit

MERIT Informed Consent Form

Participant ID: __ __ __ - __ __

Funded by NHS National Institute for Health Research

		PLEASE INITIAL
1	I confirm I have read and understood the information sheet V____ dated __ __ / __ __ __ / __ __ __ __ for the above study and have had the opportunity to ask questions and have these answered satisfactorily.	___ ___
2	I understand my participation is voluntary and that I am free to withdraw at any time, without giving any reason, and without my medical care or legal rights being affected.	___ ___
3	I understand that relevant sections of my medical notes and data collected during the study may be looked at by individuals from the University of Oxford, the relevant GP practice and from regulatory authorities, where it is relevant to my taking part in research. I give permission for these individuals to have access to my records.	___ ___
4	I agree for my identifiable information to be stored securely for up to three months after the end of the study, and research documents with my personal information (i.e. this signed Consent Form) stored securely for five years after the end of the study.	___ ___
5	I agree to my General Practitioner being informed of my participation in the study, and being sent a copy of this signed Consent Form.	___ ___
6	I agree to donate urine samples, which will be frozen and stored for further analyses relating to this study. I consider these samples a gift to the University of Oxford and I understand I will not gain any direct personal or financial benefit from them.	___ ___
7	I understand that I will be asked to complete questionnaires online using a website from the University of Oxford	___ ___
8	I agree to take part in this study.	___ ___

For peer review only

For peer review only - http://bmjopen.bmj.com/site/about/guidelines.xhtml

NUFFIELD DEPARTMENT OF PRIMARY CARE HEALTH SCIENCES UNIVERSITY OF OXFORD

merit

MERIT Informed Consent Form

Participant ID: __ __ __ - __ __

Funded by NHS National Institute for Health Research

ADDITIONAL (optional, not required for study participation)	**YES**	**NO**
I agree to donate one perineal swab sample at the beginning of the study, which will be stored for further analyses relating to this study. I consider this sample a gift to the University of Oxford and I understand I will not gain any direct personal or financial benefit from this.	__ __	__ __
I agree to being contacted about the qualitative interviews in this trial	__ __	__ __
I agree to be contacted about ethically approved research studies for which I may be suitable. I agree that my identifiable information will be stored securely for five years after the end of the study. I understand that agreeing to be contacted does not oblige me to participate in any further studies.	__ __	__ __
I agree for my de-identified urine samples to be used in future research, here or abroad, which has ethical approval. I understand this research may involve commercial organisations.	__ __	__ __

Participant:

Signed: ______________________ Name: ______________________________ Date: __ __ / __ __ / __ __ __ __

Researcher:

Signed: ______________________ Name: ______________________________ Date: __ __ / __ __ / __ __ __ __

For peer review only - http://bmjopen.bmj.com/site/about/guidelines.xhtml

STANDARD PROTOCOL ITEMS: RECOMMENDATIONS FOR INTERVENTIONAL TRIALS

SPIRIT 2013 Checklist: Recommended items to address in a clinical trial protocol and related documents*

**Section/item**	**Item No**	**Description**	**Page Number on which item is reported**
**Administrative information**			
Title	1	Descriptive title identifying the study design, population, interventions, and, if applicable, trial acronym	1
Trial registration	2a	Trial identifier and registry name. If not yet registered, name of intended registry	1
	2b	All items from the World Health Organization Trial Registration Data Set	1
Protocol version	3	Date and version identifier	1
Funding	4	Sources and types of financial, material, and other support	10
Roles and responsibilitie s	5a	Names, affiliations, and roles of protocol contributors	1
	5b	Name and contact information for the trial sponsor	1
	5c	Role of study sponsor and funders, if any, in study design; collection, management, analysis, and interpretation of data; writing of the report; and the decision to submit the report for publication, including whether they will have ultimate authority over any of these activities	14
	5d	Composition, roles, and responsibilities of the coordinating centre, steering committee, endpoint adjudication committee, data management team, and other individuals or groups overseeing the trial, if applicable (see Item 21a for data monitoring committee)	10
**Introduction**			

For peer review only

For peer review only - http://bmjopen.bmj.com/site/about/guidelines.xhtml

Background and rationale	6a	Description of research question and justification for undertaking the trial, including summary of relevant studies (published and unpublished) examining benefits and harms for each intervention	4
	6b	Explanation for choice of comparators	6
Objectives	7	Specific objectives or hypotheses	5
Trial design	8	Description of trial design including type of trial (eg, parallel group, crossover, factorial, single group), allocation ratio, and framework (eg, superiority, equivalence, noninferiority, exploratory)	5
**Methods: Participants, interventions, and outcomes**			
Study setting	9	Description of study settings (eg, community clinic, academic hospital) and list of countries where data will be collected. Reference to where list of study sites can be obtained	5
Eligibility criteria	10	Inclusion and exclusion criteria for participants. If applicable, eligibility criteria for study centres and individuals who will perform the interventions (eg, surgeons, psychotherapists)	6
Interventions	11a	Interventions for each group with sufficient detail to allow replication, including how and when they will be administered	6
	11b	Criteria for discontinuing or modifying allocated interventions for a given trial participant (eg, drug dose change in response to harms, participant request, or improving/worsening disease)	7
	11c	Strategies to improve adherence to intervention protocols, and any procedures for monitoring adherence (eg, drug tablet return, laboratory tests)	6
	11d	Relevant concomitant care and interventions that are permitted or prohibited during the trial	7
Outcomes	12	Primary, secondary, and other outcomes, including the specific measurement variable (eg, systolic blood pressure), analysis metric (eg, change from baseline, final value, time to event), method of aggregation (eg, median, proportion), and time point for each outcome. Explanation of the clinical relevance of chosen efficacy and harm outcomes is strongly recommended	5

For peer review only - http://bmjopen.bmj.com/site/about/guidelines.xhtml

Participant timeline	13	Time schedule of enrolment, interventions (including any run-ins and washouts), assessments, and visits for participants. A schematic diagram is highly recommended (see Figure)	6
Sample size	14	Estimated number of participants needed to achieve study objectives and how it was determined, including clinical and statistical assumptions supporting any sample size calculations	7
Recruitment	15	Strategies for achieving adequate participant enrolment to reach target sample size	7
**Methods: Assignment of interventions (for controlled trials)**			
Allocation:			
Sequence generation	16a	Method of generating the allocation sequence (eg, computer-generated random numbers), and list of any factors for stratification. To reduce predictability of a random sequence, details of any planned restriction (eg, blocking) should be provided in a separate document that is unavailable to those who enrol participants or assign interventions	6
Allocation concealment mechanism	16b	Mechanism of implementing the allocation sequence (eg, central telephone; sequentially numbered, opaque, sealed envelopes), describing any steps to conceal the sequence until interventions are assigned	6
Implementation	16c	Who will generate the allocation sequence, who will enrol participants, and who will assign participants to interventions	6
Blinding (masking)	17a	Who will be blinded after assignment to interventions (eg, trial participants, care providers, outcome assessors, data analysts), and how	5
	17b	If blinded, circumstances under which unblinding is permissible, and procedure for revealing a participant's allocated intervention during the trial	NA
**Methods: Data collection, management, and analysis**			

Data collection methods	18a	Plans for assessment and collection of outcome, baseline, and other trial data, including any related processes to promote data quality (eg, duplicate measurements, training of assessors) and a description of study instruments (eg, questionnaires, laboratory tests) along with their reliability and validity, if known. Reference to where data collection forms can be found, if not in the protocol	6
	18b	Plans to promote participant retention and complete follow-up, including list of any outcome data to be collected for participants who discontinue or deviate from intervention protocols	6
Data management	19	Plans for data entry, coding, security, and storage, including any related processes to promote data quality (eg, double data entry; range checks for data values). Reference to where details of data management procedures can be found, if not in the protocol	7
Statistical methods	20a	Statistical methods for analysing primary and secondary outcomes. Reference to where other details of the statistical analysis plan can be found, if not in the protocol	7
	20b	Methods for any additional analyses (eg, subgroup and adjusted analyses)	7
	20c	Definition of analysis population relating to protocol non-adherence (eg, as randomised analysis), and any statistical methods to handle missing data (eg, multiple imputation)	7
**Methods: Monitoring**			
Data monitoring	21a	Composition of data monitoring committee (DMC); summary of its role and reporting structure; statement of whether it is independent from the sponsor and competing interests; and reference to where further details about its charter can be found, if not in the protocol. Alternatively, an explanation of why a DMC is not needed	10
	21b	Description of any interim analyses and stopping guidelines, including who will have access to these interim results and make the final decision to terminate the trial	NA

For peer review only - http://bmjopen.bmj.com/site/about/guidelines.xhtml

Harms	22	Plans for collecting, assessing, reporting, and managing solicited and spontaneously reported adverse events and other unintended effects of trial interventions or trial conduct	NA
Auditing	23	Frequency and procedures for auditing trial conduct, if any, and whether the process will be independent from investigators and the sponsor	NA
**Ethics and dissemination**			
Research ethics approval	24	Plans for seeking research ethics committee/institutional review board (REC/IRB) approval	2
Protocol amendments	25	Plans for communicating important protocol modifications (eg, changes to eligibility criteria, outcomes, analyses) to relevant parties (eg, investigators, REC/IRBs, trial participants, trial registries, journals, regulators)	10
Consent or assent	26a	Who will obtain informed consent or assent from potential trial participants or authorised surrogates, and how (see Item 32)	6
	26b	Additional consent provisions for collection and use of participant data and biological specimens in ancillary studies, if applicable	NA
Confidentiality	27	How personal information about potential and enrolled participants will be collected, shared, and maintained in order to protect confidentiality before, during, and after the trial	NA
Declaration of interests	28	Financial and other competing interests for principal investigators for the overall trial and each study site	10
Access to data	29	Statement of who will have access to the final trial dataset, and disclosure of contractual agreements that limit such access for investigators	NA
Ancillary and post-trial care	30	Provisions, if any, for ancillary and post-trial care, and for compensation to those who suffer harm from trial participation	NA
Dissemination policy	31a	Plans for investigators and sponsor to communicate trial results to participants, healthcare professionals, the public, and other relevant groups (eg, via publication, reporting in results databases, or other data sharing arrangements), including any publication restrictions	2

For peer review only

	31b	Authorship eligibility guidelines and any intended use of professional writers	NA
	31c	Plans, if any, for granting public access to the full protocol, participant-level dataset, and statistical code	NA
**Appendices**			
Informed consent materials	32	Model consent form and other related documentation given to participants and authorised surrogates	NA
Biological specimens	33	Plans for collection, laboratory evaluation, and storage of biological specimens for genetic or molecular analysis in the current trial and for future use in ancillary studies, if applicable	NA

*It is strongly recommended that this checklist be read in conjunction with the SPIRIT 2013 Explanation & Elaboration for important clarification on the items. Amendments to the protocol should be tracked and dated. The SPIRIT checklist is copyrighted by the SPIRIT Group under the Creative Commons "Attribution-NonCommercial-NoDerivs 3.0 Unported" license.

For peer review only

D-mannose to prevent recurrent urinary tract infections (MERIT): protocol for a randomised controlled trial.

Journal:	*BMJ Open*
Manuscript ID	bmjopen-2020-037128.R2
Article Type:	Protocol
Date Submitted by the Author:	06-Nov-2020
Complete List of Authors:	Franssen, Marloes; University of Oxford, Nuffield Department of Orthopaedics, Rheumathology and Musculoskeletal Sciences<br>Cook, Johanna; Oxford University, Nuffield Department of Primary Care Health Sciences<br>Robinson, Jared; Oxford University, Nuffield Department of Primary Care Health Sciences<br>Williams, Nicola; Oxford University, Nuffield Department of Primary Care Health Sciences<br>Glogowska, Margaret; Oxford University, Nuffield Department of Primary Care Health Sciences<br>Yang, Yaling; University of Oxford, Nuffield Department of Primary Care Health Sciences<br>Allen, Julie; University of Oxford, Nuffield Department of Primary Care Health Sciences<br>Butler, Christopher C.; University of Oxford, Nuffield Department of Primary Care Health Sciences<br>Thomas, Nick; Windrush Medical Practice<br>Hay, Alastair; University of Bristol, Centre for Academic Primary Care<br>Moore, Michael; University of Southampton, Primary Care Medical Group<br>Hayward, Gail; Oxford University, Nuffield Department of Primary Care Health Sciences
**Primary Subject Heading**:	Urology
Secondary Subject Heading:	General practice / Family practice, Health economics, Infectious diseases
Keywords:	UROLOGY, Urinary tract infections < UROLOGY, QUALITATIVE RESEARCH

SCHOLARONE™ Manuscripts

BMJ

I, the Submitting Author has the right to grant and does grant on behalf of all authors of the Work (as defined in the below author licence), an exclusive licence and/or a non-exclusive licence for contributions from authors who are: i) UK Crown employees; ii) where BMJ has agreed a CC-BY licence shall apply, and/or iii) in accordance with the terms applicable for US Federal Government officers or employees acting as part of their official duties; on a worldwide, perpetual, irrevocable, royalty-free basis to BMJ Publishing Group Ltd ("BMJ") its licensees and where the relevant Journal is co-owned by BMJ to the co-owners of the Journal, to publish the Work in this journal and any other BMJ products and to exploit all rights, as set out in our licence.

The Submitting Author accepts and understands that any supply made under these terms is made by BMJ to the Submitting Author unless you are acting as an employee on behalf of your employer or a postgraduate student of an affiliated institution which is paying any applicable article publishing charge ("APC") for Open Access articles. Where the Submitting Author wishes to make the Work available on an Open Access basis (and intends to pay the relevant APC), the terms of reuse of such Open Access shall be governed by a Creative Commons licence – details of these licences and which Creative Commons licence will apply to this Work are set out in our licence referred to above.

Other than as permitted in any relevant BMJ Author's Self Archiving Policies, I confirm this Work has not been accepted for publication elsewhere, is not being considered for publication elsewhere and does not duplicate material already published. I confirm all authors consent to publication of this Work and authorise the granting of this licence.

For peer review only

D-mannose to prevent recurrent urinary tract infections (MERIT): protocol for a randomised controlled trial.

Running Heading: MERIT Protocol paper

Trial registration: ISRCTN 13283516

Protocol version 6.0 (17July2020)

Funder: National Institute for Health Research (NIHR) School for Primary Care Research Grant (Grant Reference No: 385)

Sponsor: Oxford University (ctrg@admin.ox.ac.uk)

Authors:

Marloes Franssen[1], Johanna Cook[2], Jared Robinson[2], Nicola Williams[2], Margaret Glogowska[2], Yaling Yang[2], Julie Allen[2], Chris C. Butler[2], Nick Thomas[3], Alastair D Hay[4], Michael Moore[5], Gail Hayward[2]

1: Nuffield Department of Orthopaedics, Rheumatology and Musculoskeletal Sciences, Oxford University, United Kingdom

2: Nuffield Department of Primary Care, Oxford University, Oxford, United Kingdom

3: GP Principal, Windrush Medical Practice, Witney; Clinical Research Specialty Lead - Primary Care (NIHR Clinical Research Network: Thames Valley and South Midlands), United Kingdom

4: Centre for Academic Primary Care, NIHR School Primary Care Research, Bristol Medical School: Population Health Sciences, Bristol, United Kingdom

5: Primary Care, Population Sciences and Medical Education, Faculty of Medicine, University of Southampton, United Kingdom

Correspondence to: Professor Gail Hayward – gail.hayward@phc.ox.ac.uk

Word count: 2828 Introduction to Discussion (excluding title page, abstract, references, tables and figures)

Number of tables: 1 Number of figures: 1

For peer review only

Abstract

Introduction: Recurrent urinary tract infections (RUTIs) have a significant negative impact on quality of life and healthcare costs. To date, daily prophylactic antibiotics are the only treatment which have been shown to help prevent RUTIs. D-mannose is a type of sugar which is believed to inhibit bacterial adherence to uroepithelial cells, and is already being used by some women in an attempt to prevent RUTIs. There is currently insufficient rigorous evidence on which to base decisions about its use. The MERIT study will evaluate whether D-mannose is clinically and cost effective in reducing frequency of infection and symptom burden for women presenting to UK primary care with recurrent UTI.

Methods and analysis: MERIT will be a two arm, individually randomised, double blind placebo controlled, pragmatic trial. Participants will be randomised to take D-mannose powder or placebo powder daily for six months. The primary outcome will be the number of medical attendances attributable to symptoms of RUTI. With 508 participants we will have 90% power to detect a 50% reduction in the chance of a further clinically suspected UTI, assuming 20% loss to follow up. Secondary outcomes will include: number of days of moderately bad symptoms of UTI; time to next consultation; number of clinically suspected UTIs; number of microbiologically proven UTIs; number of antibiotic courses for UTI; quality of life and healthcare utilisation related to UTI. A within trial economic evaluation will be conducted to examine cost-effectiveness of D-mannose in comparison with placebo. A nested qualitative study will explore participants' experiences and perceptions of recruitment to, and participation in a study requiring a daily treatment.

Ethics and dissemination: Ethical approval has been obtained from South West – Central Bristol Research Ethics Committee. Publication of the MERIT study is anticipated to occur in 2021.

Trial Registration: ISRCTN 13283516

Keywords: UTI recurrence, RCT, Primary care, D-Mannose, cost effectiveness, qualitative interview

For peer review only

Strengths and limitations:

- Based on current literature this will be the first large publicly funded randomised controlled trial of D-mannose for prophylaxis of recurrent urinary tract infections.
- This study is the first to use a placebo control in evaluating the benefit of D-mannose
- Obtaining the primary outcome by medical notes review will ensure data completeness
- The trial may not be powered to detect a secondary outcome of symptom burden which is also of value to patient decision making
- Although participants report weekly on their study product usage there are no objective measures available to confirm accuracy of reporting

For peer review only

1. BACKGROUND

Urinary tract infection (UTI) is the most common bacterial infection that women consult for in UK primary care (1, 2). Approximately 40-50% of women experience one UTI episode during their lives (3). Recurrent UTIs (RUTIs) have a considerable negative impact on quality of life, which extends beyond the unpleasant symptoms to distressing and disrupted sexual relationships, persistent unmanageable pain and systemic illness (4). UTI accounts for an important proportion of health care costs as a result of outpatient visits, diagnostic tests and prescriptions (5). In 2007, UTI recurrence accounted for 10.5 million outpatient consultations and 2–3 million emergency department visits in the USA alone. In addition, UTIs are the most common cause of infection in hospitalized patients, accounting for 17.2% of all nosocomial infections in England. Furthermore, UTIs result in considerable patient morbidity and time off work; hence, the management of this condition incurs large financial costs, estimated at $3.5 billion in the USA per year (6).

A systematic review of randomised controlled trials identified antibiotic prophylaxis as the only treatment which has been demonstrated to help prevent RUTIs. Antibiotics taken daily for six to twelve months were more effective than placebo at preventing recurrent infection (7), and national guidelines advocate their use for this indication (8). However, antibiotics also resulted in more severe and unpleasant side effects (e.g. vomiting, urticaria, candidiasis). Furthermore, once antibiotic prophylaxis is discontinued, even after extended periods, approximately 50-60% of women will experience a further UTI within three months (9, 10). Thus, antibiotic prophylaxis does not exert benefit once stopped, and is directly linked to antibiotic resistance in uropathogens (11). Antibiotic resistance has been associated with an increased duration of severe symptoms of UTIs, irrespective of the use of an appropriate antibiotic (2, 11).

D-mannose is a type of sugar (a monosaccharide isomer of glucose), which is thought to inhibit bacterial adherence to uroepithelial cells by binding to a site on the tip of the fimbria (12) and has shown benefit in animal models in preventing UTIs (13).

Currently D-mannose is available commercially to the public as a food supplement, and is favoured by many women who have RUTIs, but until recently, there has been little empirical evidence to support its use. An open label randomised three arm trial including 308 women with RUTI seen in outpatient settings (14) found that daily use of D-mannose for six months resulted in an absolute reduction in incidence of further UTI of 45% from a proportion of 60% in the usual care arm, with no adverse events. The proportion of women experiencing a RUTI over six months was reduced by 11% compared to daily antibiotic use. This finding is supported by recent smaller studies (15-18).

Although there are indicators of efficacy from small underpowered trials, the only adequately powered study to date (15) was not placebo controlled and found an unexpectedly high RUTI incidence in the control arm. Furthermore, a microbiologically confirmed UTI was a requirement for entry to the study, and participants were withdrawn once they developed a UTI on treatment. Therefore, true incidence of UTI could not be established, a measure for women who experience frequent RUTIs, who are also the most likely candidates for prophylaxis. Finally, all women on hormonal contraception were excluded, which may reduce applicability to the women at high risk of RUTI.

For peer review only

D-mannose is found naturally in small quantities in numerous food sources, such as coffee, baker's yeast, egg white, fruits such as apples, cranberries and mangos, and also in legumes such as soybeans, kidney beans and peanuts (19). It is absorbed in the upper gastrointestinal tract and excreted in the urine (14).

D-mannose may offer an alternative to antibiotic prophylaxis in women who experience RUTI and in turn to contribute to better antimicrobial stewardship in primary care. However, the current evidence base is inadequate to help women with RUTI to make informed decisions about the use of D-mannose prophylaxis. The high costs (at least £25 a month) associated with its purchase add weight to the need to establish whether GPs should advise their patients to use D-mannose for this indication.

The MERIT double blind placebo-controlled randomised controlled trial aims to evaluate the effectiveness of D-mannose in women suffering with RUTI presenting to UK primary care and its cost effectiveness.

2. METHODS AND DESIGN

2.1 Study aims, research questions and outcomes

The primary aim of MERIT is to assess the effectiveness of daily use of D-mannose compared with placebo in preventing symptomatic UTI in women.

The primary outcome of the trial will be the proportion of women experiencing at least one further episode of clinically suspected UTI for which they contact ambulatory care (out of hours primary care, in hours primary care, ambulance or the emergency department) within six months of study entry.

Secondary outcomes will include (within six months of study entry):

-Number of days of moderately bad (or worse) symptoms of UTI

-Time to next consultation with a clinically suspected UTI

-Number of clinically suspected UTIs

-Number of microbiologically proven UTIs

-Number of antibiotic courses for UTI

-Report of consumption of antibiotics using diary during periods of infection

-Proportion of women with a resistant uropathogen culture during an episode of acute infection

-Hospital admissions related to UTI

-Quality of life and healthcare utilisation related to UTI

-Healthcare utilisation recorded in the participant diary and during a notes review

-Acceptability and process evaluation conducted via telephone interviews (after 6 months)

For peer review only

2.2 Study design and setting

A two arm, individually randomised, double blind placebo controlled, pragmatic trial. At least 50 GP practices in England and Wales will be invited to take part in the trial. Recruitment will run from March 2019 until January 2020.

2.3 Eligibility

This trial will recruit female participants over 18 years with a primary care clinical record of having presented to ambulatory care with RUTIs three or more times in the last year or two or more times in the last six months. Exclusion criteria are: participants who are pregnant, lactating or planning pregnancy during the course of the study; formal diagnosis of interstitial cystitis or overactive bladder syndrome; prophylactic antibiotics started in the last three months and unwilling to discontinue, or intention to start in the next six months; currently using D-mannose and unwilling to discontinue for the duration of the study; nursing home resident; catheterised, including intermittent self-catheterisation; use of Uromune; participation in a research study involving an investigational product in the past twelve weeks.

2.4 Baseline assessment

Participants will have a baseline assessment (either in person with their GP or research nurse online, or by telephone with a member of the research team). During the baseline assessment the study will be explained, informed consent (see supplementary files) will be obtained and data will be collected (see table 1). Participants will also be asked to send in a urine sample (when they are asymptomatic of a UTI) at baseline and have the option to also send in a perineal swab sample.

Table 1: Data collection throughout the trial

Baseline:
1. Demographic questions: including age,
2. Past medical history [by patients]
3. Use of contraceptives and hormonal treatment
4. UTI episodes in the last 12 months
5. EQ-5D-5L
Weekly contact/Monthly contact:
1. UTI episodes in last week/month respectively
Daily UTI diary
1. UTI symptoms
2. UTI treatment
3. EQ-5D-5L

For peer review only

Six month questionnaire
1. UTI episodes in the last month
2. EQ-5D-5L
Notes review and urine culture result
1. Recorded UTIs during the study period
2. Healthcare contact for UTIs recorded
3. Antibiotics given for UTIs recorded
4. Culture results for UTIs recorded
5. Unscheduled hospital admissions

2.5 Randomisation

After the baseline assessment, participants will be randomised by a member of the research team using a validated internet based randomisation system with an emergency randomisation list available. Randomisation will use variable block sizes and will be stratified by GP practice ensuring a balance of the two arms within each practice.

2.6 Intervention and placebo groups

Placebo will consist of two grams of a sugar powder which is similar in texture and taste to D-mannose but fully absorbed by the liver to be taken daily for six months.

Intervention will consist of two grams of D-mannose powder to be taken daily for six months.

An adequate supply of the study product will be sent directly from the research team to the participant after randomisation, after two months and after four months.

2.7 Follow up

All participants will be asked to complete short weekly questionnaires, sent to them via text or email; they also will have the option of completing them telephonically directly with the research team. The weekly questionnaire will collect the information of participant's adherence to study medication, and whether the participant has had any symptomatic UTI episodes. Participants will also be contacted monthly by phone by the research team to complete a monthly questionnaire which is similar to the weekly one if there are two or more weekly questionnaires not being completed. They will also be asked to complete a daily UTI symptom diary if they experience a UTI. The information will be collected via weekly and monthly contact if they fail to complete the symptom diary although they experience one. During a UTI they will be asked to send the lab a urine sample, alongside any sample they might provide to their GP. They will also be asked for a further urine sample two days after symptoms have resolved. See table 1 for details. Primary care electronic medical record reviews will be conducted to collect UTI related health care contacts, culture results, and prescriptions during

For peer review only

the following up period. Participants will receive a £10 voucher after every two months of participation (£30 pounds in total). See figure 1 for the participant flow through the trial.

2.8 Sample size considerations

A recent study to evaluate prophylactic treatment for RUTI in a similar population (20) found that 26.6% of women in the control arm experienced a RUTI within six months. Our patient and public involvement advisors suggested that in order to commit to daily use of a prophylactic regime, they would require evidence of at least a 50% reduction in the chance of a further UTI during the period of prophylaxis. To detect this reduction with a 2-sided Fisher's Exact test with 90% power and an alpha of 0.05 we would require 203 participants in each arm. This equates to 508 participants if a 20% loss to follow up is assumed. This sample size is also adequate to power the key secondary outcome (the number of RUTI's experienced over six months), and detect a relative incidence rate of 0.5 between the treatment and placebo groups, assuming a base rate of 0.36 as estimated by Maki et al. (2016) (20). If the estimated percentage of participants who have either withdrawn or failed to respond to any study team communication for an extended period seems likely to rise above the 20% initially allowed for, we will recruit additional participants, up to a maximum of 598 participants.

2.9 Statistical analysis

The primary outcome, the proportion of women experiencing at least one further episode of UTI symptoms for which they visited their GP within six months of study entry, and other binary outcomes, will be analysed on an intention-to-treat basis by means of a generalised linear mixed effects model with binomial distribution and log link function, including a random effect for practice and fixed effect for randomisation group. Therefore, treatment groups will be compared on the basis of an adjusted risk ratio. The number of days of moderately bad symptoms of UTI, the number of UTI's experienced in six months, and number of antibiotic courses for UTI in six months, will be analysed by means of a generalised linear mixed effects model using the Poisson distribution and log link function, including a random effect for practice and a fixed effect for randomised group. Defined Daily Doses (DDDs) will be analysed by means of a linear mixed effects model including a random effect for practice and fixed effects for randomised group and baseline DDD, treating this outcome as continuous. We will analyse the overall DDD as well as the individual antibiotic DDDs.

The amount of missing primary outcome data is expected to be very low as it is collected via notes review. The model chosen to analyse the primary outcome implicitly accounts for data missing at random, however the data missing mechanism will be explored. Summary statistics will be presented for baseline covariates of those participants who completed and those who were lost to follow-up for the primary outcome. Baseline covariates associated with missingness will be identified by analysing each baseline covariate in a logistic regression model to determine which (if any) are associated with missingness of the primary outcome. The associated P-value will be reported alongside the summary statistics. Any baseline factors found to be associated with missingness of the primary outcome will be included in a sensitivity analysis.

2.10 Data management

For peer review only

Data Management will be performed in accordance with Primary Care Clinical Trials Unit Data Management standard operating procedures. Study specific procedures will be outlined in a Data Management Plan to ensure that high quality data are produced for statistical analysis.

2.11 Potential Risks

It is anticipated that the potential risks of this study are low and similar to those attributable to usual care.

2.12 Health economic evaluation

A cost effectiveness analysis from a health system perspective with a time horizon of six months will be conducted alongside this study. The primary outcome measure for the cost utility analysis will be the quality adjusted life years (QALYs). Data collection to facilitate analysis includes resource use and health outcomes. Data from the participant diary and electronic medical record review will be the main source of resource use. Unit costs associated with resource use items will be obtained from national standards. Health outcomes will be measured using the 5-level version of the EQ-5D questionnaire (EQ-5D-5L).

Data analysis will be conducted on an intention-to-treat basis using an incremental approach. Resource use and unit cost will be combined to calculate health care costs for each participant and mean cost for each study arm. EQ-5D-5L utility values will be calculated using the UK-based algorithm. Using the under the curve methods to combine utility values and associated time durations will produce QALYs for each participant and mean QALYs for each study arm during the six month study period. Mean differences in costs and QALYs between the study arms will be estimated as incremental cost per QALY gained. Given the fact that antibiotics are currently the mainstay treatment for both acute and recurrent UTIs, the issue of how the cost of antibiotic resistance should be incorporated into economic evaluation will be explored in the analysis.

2.13 Nested Qualitative study

We will recruit a maximum variation sample of 35 participants across both study arms for the nested qualitative study, continuing recruitment until data saturation is reached. A balanced list of participants will be drawn up for the qualitative researchers. The topic guide will include participants' experiences and perceptions of recruitment to, and participation in a study that requires taking a daily study product (whether D-mannose or placebo), exploring the level of perceived benefit patients anticipate would be required for them to continue this type of regimem, and facilitators and barriers to adhering to prophylactic treatment. For participants' convenience, interviews will be conducted by telephone. Thematic analysis of the interviews will take into account issues identified from the literature and clinical research context, as well as inductively allowing new themes and ideas to emerge from the data. Analysis will be guided by the constant comparative method (21), which will include reading and familiarisation with the transcripts, noting and recording initial themes and then conducting systematic and detailed open coding using NVivo12 (22), a qualitative data analysis software. Analysis will proceed in an iterative manner – thus, the coding of a first set of interviews will generate an initial coding framework, which will be further developed and refined as further interviews are conducted and analysis proceeds. The researcher will draw on the clinical expertise of the rest of the research team in developing the

For peer review only

coding framework and critically discussing ideas for categories emerging from the data, to ensure trustworthiness. A reflexive journal will assist in interpreting data and forming conclusions.

2.14 Patient and Public Involvement

Members of the public were involved in the design of the trial, reviewed patient facing documents and they will be active members of the trial steering committee.

3. DISCUSSION

The MERIT Study will be the first large, publicly funded, double blind randomised trial of the clinical and cost effectiveness of daily D-Mannose for preventing RUTI in primary care. This overview of the protocol describes the plans for a pragmatic study recruiting women who suffer from recurrent UTI recruited in UK primary care. This study will fill a major gap in the evidence base about whether women with recurrent UTIs should initiate or continue to use this food supplement to prevent RUTI. If D-mannose is proven to be effective for the treatment of RUTIs this could benefit affected women and also contribute to antimicrobial stewardship. On the other hand, if found to be ineffective, costs spent on an ineffective intervention will be saved and attention can be refocussed on other, perhaps more effective prophylactic approaches, as well as redirected research efforts.

4. Ethics and Dissemination

Ethical approval has been obtained from South West – Central Bristol Research Ethics Committee (reference: 18/SW/0245). Any subsequent protocol amendments will be agreed with both sponsor and ethics committee prior to implementation. Publication of the MERIT study is anticipated to occur in 2021.

Supplementary files:

- Figure 1: Participant flow through the trial
- Example of participant consent form

LIST OF ABBREVIATIONS

DDD: defined daily dosage; EQ-5D-5L: 5 level version of the EQ-5D questionnaire; GP: General Practitioner; RUTIs: recurrent urinary tract infections; QALY: quality adjusted life years; UTI: Urinary Tract Infection;

DECLARATIONS

Ethics approval and consent to participate

Research ethics approval was obtained by the South West- Central Bristol Research Ethics Committee. The study sponsor reviewed and ensured all indemnity and insurance requirements for the trial were in place prior to the start of recruitment. Participants will provide written informed consent prior to enrolment. Site specific approval has been obtained from the Thames Valley and South Midlands as a whole, and locally from all relevant Primary Care Organisations. An independent trial steering group will monitor study progress assisted by an independent data monitoring committee will periodically review the study.

For peer review only

Consent for publication

Not applicable

Availability of data and material

Not applicable as this is as study protocol and does not contain data or results.

Competing interests

None to declare

Funding

The trial is funded by a School for Primary Care research grant. Service support costs are administered through the NIHR Clinical Research Network: Thames Valley and South Midlands

This article presents independent research commissioned by the National Institute for Health Research (NIHR) under a School for Primary Care Research Grant (reference number:385). The views expressed in this publication are those of the authors and not necessarily those of the NHS, the NIHR or the Department of Health.

Authors contributions

GH had the original idea with CCB and they designed the study and obtained the funding. MF and JC wrote the first draft, NW provided the statistical section. YY provided the health economic section, MG provided the qualitative section. All authors (JR, JA, NT, AH, MM) subsequently critically edited the manuscript. GH will be guarantor for the manuscript.

Dissemination

The results of the MERIT will be published in peer-reviewed journals, in addition to being presented at conferences. It is anticipated that the results will be published in 2021, after the six month follow-up of all recruited participants.

Acknowledgements

The authors acknowledge the support of the Primary Care Clinical Trials Unit. Patient representatives are Sylvia Bailey and Valerie Tate. Additional members of the TSC are Rebecca Cannings-John (chair), Laura Shallcross and Akke Vellinga. The sponsor and funder had no role in the study design; collection, management, analysis, and interpretation of data, writing of the report; or the decision to submit the report for publication, which was made jointly by the authors who have all approved the final manuscript.

For peer review only

For peer review only

REFERENCES

1. Butler CC, Hillier S, Roberts Z, Dunstan F, Howard A, Palmer S. Antibiotic-resistant infections in primary care are symptomatic for longer and increase workload: outcomes for patients with E. coli UTIs. Br J Gen Pract. 2006;56(530):686-92.
2. Little P, Moore MV, Turner S, Rumsby K, Warner G, Lowes JA, et al. Effectiveness of five different approaches in management of urinary tract infection: randomised controlled trial. BMJ. 2010;340:c199.
3. Stapleton A. Host factors in susceptibility to urinary tract infections. Adv Exp Med Biol. 1999;462:351-8.
4. Flower A, Bishop FL, Lewith G. How women manage recurrent urinary tract infections: an analysis of postings on a popular web forum. BMC Fam Pract. 2014;15:162.
5. Butler CC, Hawking MK, Quigley A, McNulty CA. Incidence, severity, help seeking, and management of uncomplicated urinary tract infection: a population-based survey. Br J Gen Pract. 2015;65(639):e702-7.
6. Sihra N, Goodman A, Zakri R, Sahai A, Malde S. Nonantibiotic prevention and management of recurrent urinary tract infection. Nat Rev Urol. 2018;15(12):750-76.
7. Albert X, Huertas I, Pereiro, II, Sanfelix J, Gosalbes V, Perrota C. Antibiotics for preventing recurrent urinary tract infection in non-pregnant women. Cochrane Database Syst Rev. 2004(3):CD001209.
8. SIGN. Management of suspected bacterial urinary tract infection in adults 2012 [Available from: http://www.sign.ac.uk.
9. Car J, Sheikh A. Recurrent urinary tract infection in women. BMJ. 2003;327(7425):1204.
10. Harding GK, Ronald AR, Nicolle LE, Thomson MJ, Gray GJ. Long-term antimicrobial prophylaxis for recurrent urinary tract infection in women. Rev Infect Dis. 1982;4(2):438-43.
11. Costelloe C, Metcalfe C, Lovering A, Mant D, Hay AD. Effect of antibiotic prescribing in primary care on antimicrobial resistance in individual patients: systematic review and meta-analysis. BMJ. 2010;340:c2096.
12. Bouckaert J, Berglund J, Schembri M, De Genst E, Cools L, Wuhrer M, et al. Receptor binding studies disclose a novel class of high-affinity inhibitors of the Escherichia coli FimH adhesin. Mol Microbiol. 2005;55(2):441-55.
13. Michaels EK, Chmiel JS, Plotkin BJ, Schaeffer AJ. Effect of D-mannose and D-glucose on Escherichia coli bacteriuria in rats. Urol Res. 1983;11(2):97-102.
14. Sharma V, Ichikawa M, Freeze HH. Mannose metabolism: more than meets the eye. Biochem Biophys Res Commun. 2014;453(2):220-8.
15. Kranjcec B, Papes D, Altarac S. D-mannose powder for prophylaxis of recurrent urinary tract infections in women: a randomized clinical trial. World J Urol. 2014;32(1):79-84.
16. Xing Hu YS, Peng Zhang, Ming Miao, Toa Zhang, Bo Jiang. D-Mannose: Properties, Production, and Applications: An Overview. Comprehensive Reviews in Food Science and Food Safety. 2016;15:773-85.
17. Domenici L, Monti M, Bracchi C, Giorgini M, Colagiovanni V, Muzii L, et al. D-mannose: a promising support for acute urinary tract infections in women. A pilot study. Eur Rev Med Pharmacol Sci. 2016;20(13):2920-5.
18. Kuzmenko AV, Kuzmenko VV, Gyaurgiev TA. [Efficacy of combined antibacterial-prebiotic therapy in combination with D-mannose in women with uncomplicated lower urinary tract infection]. Urologiia. 2019(6):38-43.
19. Xing Hu ea. d-Mannose: Properties, production and Applications: An Overview.
20. Maki KC, Kaspar KL, Khoo C, Derrig LH, Schild AL, Gupta K. Consumption of a cranberry juice beverage lowered the number of clinical urinary tract infection episodes in women with a recent history of urinary tract infection. Am J Clin Nutr. 2016;103(6):1434-42.
21. Silverman DT. Doing Qualitative Research. Fifth Edition ed: Sage: London; 2017.

For peer review only

22. International Q. NVivo [Available from: https://www.qsrinternational.com/nvivo/what-is-nvivo/nvivo-12-promo.

For peer review only

Figure 1: Flow through the trial

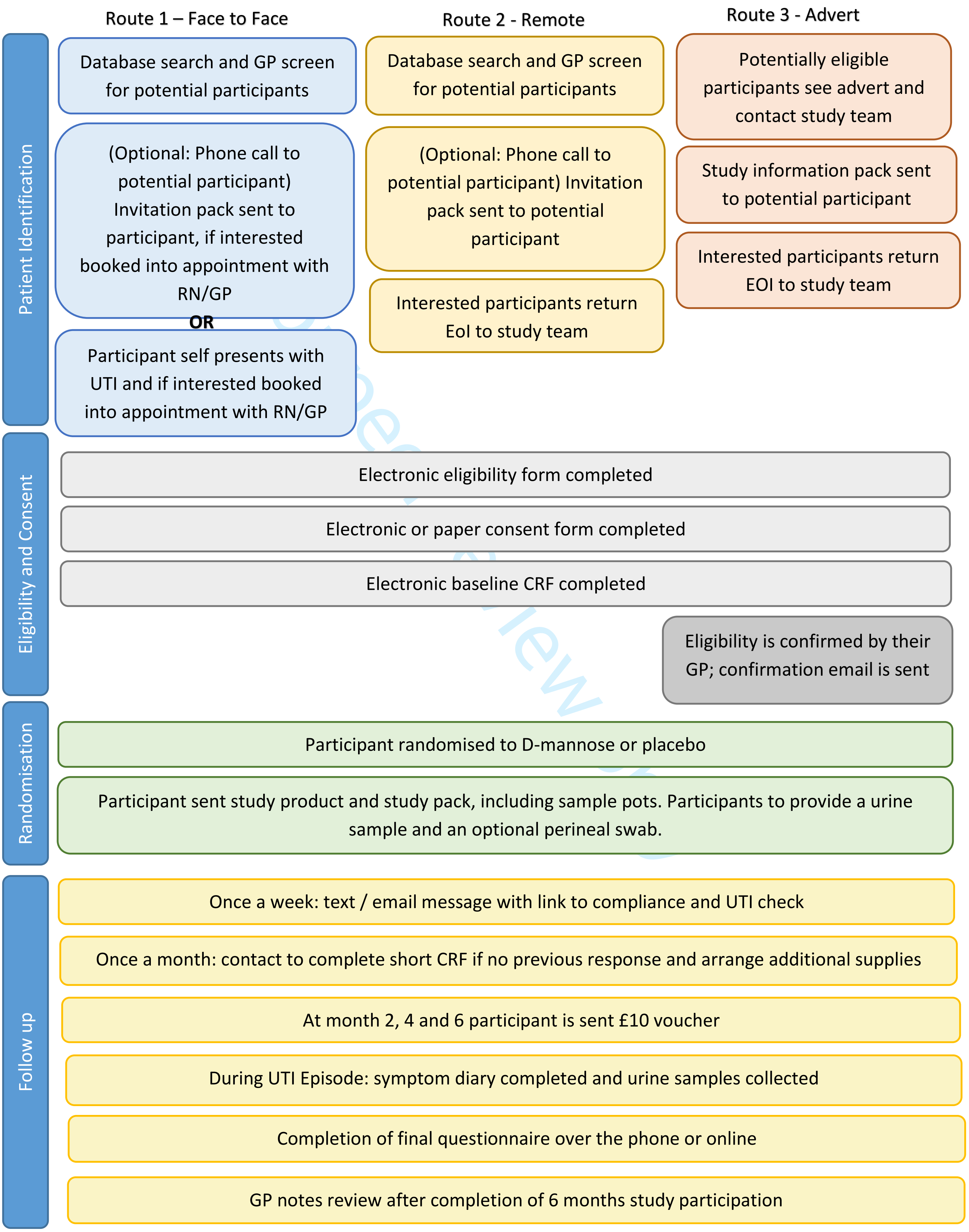

NUFFIELD DEPARTMENT OF PRIMARY CARE HEALTH SCIENCES
UNIVERSITY OF OXFORD

merit

MERIT Informed Consent Form

Participant ID: __ __ __ - __ __

Funded by NHS National Institute for Health Research

		PLEASE INITIAL
1	I confirm I have read and understood the information sheet V____ dated __ __ / __ __ __ / __ __ __ __ for the above study and have had the opportunity to ask questions and have these answered satisfactorily.	___ ___
2	I understand my participation is voluntary and that I am free to withdraw at any time, without giving any reason, and without my medical care or legal rights being affected.	___ ___
3	I understand that relevant sections of my medical notes and data collected during the study may be looked at by individuals from the University of Oxford, the relevant GP practice and from regulatory authorities, where it is relevant to my taking part in research. I give permission for these individuals to have access to my records.	___ ___
4	I agree for my identifiable information to be stored securely for up to three months after the end of the study, and research documents with my personal information (i.e. this signed Consent Form) stored securely for five years after the end of the study.	___ ___
5	I agree to my General Practitioner being informed of my participation in the study, and being sent a copy of this signed Consent Form.	___ ___
6	I agree to donate urine samples, which will be frozen and stored for further analyses relating to this study. I consider these samples a gift to the University of Oxford and I understand I will not gain any direct personal or financial benefit from them.	___ ___
7	I understand that I will be asked to complete questionnaires online using a website from the University of Oxford	___ ___
8	I agree to take part in this study.	___ ___

For peer review only

For peer review only - http://bmjopen.bmj.com/site/about/guidelines.xhtml

NUFFIELD DEPARTMENT OF PRIMARY CARE HEALTH SCIENCES
UNIVERSITY OF OXFORD
merit

MERIT Informed Consent Form

Participant ID: __ __ __ - __ __

Funded by NHS National Institute for Health Research

ADDITIONAL (optional, not required for study participation)	**YES**	**NO**
I agree to donate one perineal swab sample at the beginning of the study, which will be stored for further analyses relating to this study. I consider this sample a gift to the University of Oxford and I understand I will not gain any direct personal or financial benefit from this.	__ __	__ __
I agree to being contacted about the qualitative interviews in this trial	__ __	__ __
I agree to be contacted about ethically approved research studies for which I may be suitable. I agree that my identifiable information will be stored securely for five years after the end of the study. I understand that agreeing to be contacted does not oblige me to participate in any further studies.	__ __	__ __
I agree for my de-identified urine samples to be used in future research, here or abroad, which has ethical approval. I understand this research may involve commercial organisations.	__ __	__ __

Participant:

Signed: ______________________ Name: ______________________________ Date: __ __ / __ __ / __ __ __ __

Researcher:

Signed: ______________________ Name: ______________________________ Date: __ __ / __ __ / __ __ __ __

For peer review only - http://bmjopen.bmj.com/site/about/guidelines.xhtml

SPIRIT

STANDARD PROTOCOL ITEMS: RECOMMENDATIONS FOR INTERVENTIONAL TRIALS

SPIRIT 2013 Checklist: Recommended items to address in a clinical trial protocol and related documents*

Section/item	Item No	Description	Page Number on which item is reported
**Administrative information**			
Title	1	Descriptive title identifying the study design, population, interventions, and, if applicable, trial acronym	1
Trial registration	2a	Trial identifier and registry name. If not yet registered, name of intended registry	1
	2b	All items from the World Health Organization Trial Registration Data Set	1
Protocol version	3	Date and version identifier	1
Funding	4	Sources and types of financial, material, and other support	10
Roles and responsibilities	5a	Names, affiliations, and roles of protocol contributors	1
	5b	Name and contact information for the trial sponsor	1
	5c	Role of study sponsor and funders, if any, in study design; collection, management, analysis, and interpretation of data; writing of the report; and the decision to submit the report for publication, including whether they will have ultimate authority over any of these activities	14
	5d	Composition, roles, and responsibilities of the coordinating centre, steering committee, endpoint adjudication committee, data management team, and other individuals or groups overseeing the trial, if applicable (see Item 21a for data monitoring committee)	10
**Introduction**			

For peer review only

Background and rationale	6a	Description of research question and justification for undertaking the trial, including summary of relevant studies (published and unpublished) examining benefits and harms for each intervention	4
	6b	Explanation for choice of comparators	6
Objectives	7	Specific objectives or hypotheses	5
Trial design	8	Description of trial design including type of trial (eg, parallel group, crossover, factorial, single group), allocation ratio, and framework (eg, superiority, equivalence, noninferiority, exploratory)	5
**Methods: Participants, interventions, and outcomes**			
Study setting	9	Description of study settings (eg, community clinic, academic hospital) and list of countries where data will be collected. Reference to where list of study sites can be obtained	5
Eligibility criteria	10	Inclusion and exclusion criteria for participants. If applicable, eligibility criteria for study centres and individuals who will perform the interventions (eg, surgeons, psychotherapists)	6
Interventions	11a	Interventions for each group with sufficient detail to allow replication, including how and when they will be administered	6
	11b	Criteria for discontinuing or modifying allocated interventions for a given trial participant (eg, drug dose change in response to harms, participant request, or improving/worsening disease)	7
	11c	Strategies to improve adherence to intervention protocols, and any procedures for monitoring adherence (eg, drug tablet return, laboratory tests)	6
	11d	Relevant concomitant care and interventions that are permitted or prohibited during the trial	7
Outcomes	12	Primary, secondary, and other outcomes, including the specific measurement variable (eg, systolic blood pressure), analysis metric (eg, change from baseline, final value, time to event), method of aggregation (eg, median, proportion), and time point for each outcome. Explanation of the clinical relevance of chosen efficacy and harm outcomes is strongly recommended	5

For peer review only - http://bmjopen.bmj.com/site/about/guidelines.xhtml

Participant timeline	13	Time schedule of enrolment, interventions (including any run-ins and washouts), assessments, and visits for participants. A schematic diagram is highly recommended (see Figure)	6
Sample size	14	Estimated number of participants needed to achieve study objectives and how it was determined, including clinical and statistical assumptions supporting any sample size calculations	7
Recruitment	15	Strategies for achieving adequate participant enrolment to reach target sample size	7
**Methods: Assignment of interventions (for controlled trials)**			
Allocation:			
Sequence generation	16a	Method of generating the allocation sequence (eg, computer-generated random numbers), and list of any factors for stratification. To reduce predictability of a random sequence, details of any planned restriction (eg, blocking) should be provided in a separate document that is unavailable to those who enrol participants or assign interventions	6
Allocation concealment mechanism	16b	Mechanism of implementing the allocation sequence (eg, central telephone; sequentially numbered, opaque, sealed envelopes), describing any steps to conceal the sequence until interventions are assigned	6
Implementation	16c	Who will generate the allocation sequence, who will enrol participants, and who will assign participants to interventions	6
Blinding (masking)	17a	Who will be blinded after assignment to interventions (eg, trial participants, care providers, outcome assessors, data analysts), and how	5
	17b	If blinded, circumstances under which unblinding is permissible, and procedure for revealing a participant's allocated intervention during the trial	NA
**Methods: Data collection, management, and analysis**			

Data collection methods	18a	Plans for assessment and collection of outcome, baseline, and other trial data, including any related processes to promote data quality (eg, duplicate measurements, training of assessors) and a description of study instruments (eg, questionnaires, laboratory tests) along with their reliability and validity, if known. Reference to where data collection forms can be found, if not in the protocol	6
	18b	Plans to promote participant retention and complete follow-up, including list of any outcome data to be collected for participants who discontinue or deviate from intervention protocols	6
Data management	19	Plans for data entry, coding, security, and storage, including any related processes to promote data quality (eg, double data entry; range checks for data values). Reference to where details of data management procedures can be found, if not in the protocol	7
Statistical methods	20a	Statistical methods for analysing primary and secondary outcomes. Reference to where other details of the statistical analysis plan can be found, if not in the protocol	7
	20b	Methods for any additional analyses (eg, subgroup and adjusted analyses)	7
	20c	Definition of analysis population relating to protocol non-adherence (eg, as randomised analysis), and any statistical methods to handle missing data (eg, multiple imputation)	7
**Methods: Monitoring**			
Data monitoring	21a	Composition of data monitoring committee (DMC); summary of its role and reporting structure; statement of whether it is independent from the sponsor and competing interests; and reference to where further details about its charter can be found, if not in the protocol. Alternatively, an explanation of why a DMC is not needed	10
	21b	Description of any interim analyses and stopping guidelines, including who will have access to these interim results and make the final decision to terminate the trial	NA

Harms	22	Plans for collecting, assessing, reporting, and managing solicited and spontaneously reported adverse events and other unintended effects of trial interventions or trial conduct	NA
Auditing	23	Frequency and procedures for auditing trial conduct, if any, and whether the process will be independent from investigators and the sponsor	NA
**Ethics and dissemination**			
Research ethics approval	24	Plans for seeking research ethics committee/institutional review board (REC/IRB) approval	2
Protocol amendments	25	Plans for communicating important protocol modifications (eg, changes to eligibility criteria, outcomes, analyses) to relevant parties (eg, investigators, REC/IRBs, trial participants, trial registries, journals, regulators)	10
Consent or assent	26a	Who will obtain informed consent or assent from potential trial participants or authorised surrogates, and how (see Item 32)	6
	26b	Additional consent provisions for collection and use of participant data and biological specimens in ancillary studies, if applicable	NA
Confidentiality	27	How personal information about potential and enrolled participants will be collected, shared, and maintained in order to protect confidentiality before, during, and after the trial	NA
Declaration of interests	28	Financial and other competing interests for principal investigators for the overall trial and each study site	10
Access to data	29	Statement of who will have access to the final trial dataset, and disclosure of contractual agreements that limit such access for investigators	NA
Ancillary and post-trial care	30	Provisions, if any, for ancillary and post-trial care, and for compensation to those who suffer harm from trial participation	NA
Dissemination policy	31a	Plans for investigators and sponsor to communicate trial results to participants, healthcare professionals, the public, and other relevant groups (eg, via publication, reporting in results databases, or other data sharing arrangements), including any publication restrictions	2

For peer review only

	31b	Authorship eligibility guidelines and any intended use of professional writers	NA
	31c	Plans, if any, for granting public access to the full protocol, participant-level dataset, and statistical code	NA
**Appendices**			
Informed consent materials	32	Model consent form and other related documentation given to participants and authorised surrogates	NA
Biological specimens	33	Plans for collection, laboratory evaluation, and storage of biological specimens for genetic or molecular analysis in the current trial and for future use in ancillary studies, if applicable	NA

*It is strongly recommended that this checklist be read in conjunction with the SPIRIT 2013 Explanation & Elaboration for important clarification on the items. Amendments to the protocol should be tracked and dated. The SPIRIT checklist is copyrighted by the SPIRIT Group under the Creative Commons "Attribution-NonCommercial-NoDerivs 3.0 Unported" license.

For peer review only
